# Theoretical proposal of a low-loss wide-bandwidth silicon photonic crystal fiber for supporting 30 orbital angular momentum modes

**DOI:** 10.1371/journal.pone.0189660

**Published:** 2017-12-13

**Authors:** Xun Xu, Hongzhi Jia, Yu Lei, Chunhua Jia, Gang Liu, Junyu Chai, Yanting Peng, Jilong Xie

**Affiliations:** Engineering Research Center of Optical Instruments and Systems, Ministry of Education, Shanghai Key Laboratory of Modern Optical Systems, School of Optical-Electrical and Computer Engineering, University of Shanghai for Science and Technology, Shanghai, China; Bar-Ilan University, ISRAEL

## Abstract

We propose a novel four-ring hollow-core silicon photonic crystal fiber (PCF), and we systematically and theoretically investigate the properties of their vector modes. Our PCF can stably support 30 OAM states from the wavelength of 1.5 μm to 2.4 μm, with a large effective refractive index separation of above 1×10^−4^. The confinement loss is less than 1×10^−9^ dB/m at the wavelength of 1.55 μm, and the average confinement loss is less than 1×10^−8^ dB/m from the wavelength of 1.2 μm to 2.4 μm. Moreover, the curve of the dispersion tends to flatten as the wavelength increases. In addition, we comparably investigate PCFs with different hole spacing. This kind of fiber structure will be a potential candidate for high-capacity optical fiber communications and OAM sensing applications using fibers.

## Introduction

Spatial beams carrying orbital angular momentum (OAM) are depicted by a helical phase term of exp(*ilφ*), where *l* is the topological charge number and *φ* represents the azimuthal angle [1,2]. OAM beams have witnessed extensive applications such as photo entanglement [3,4], optical tweezing [2], nanoscale microscope [5], image [6], and high-capacity optical communication [7–9], owing to their spiral phase front field profile. The OAM modes multiplexing technology, which is based on the orthogonality between any two OAM modes and the theoretically boundless states of the modes, especially possesses an enormous potential in the field of short-distance, high-capacity optical fiber communication. This technology might be a good solution to the currently deficient state of data capacity [8,10]. With regard to data transmission, a sufficient number of OAM modes, which is proportional to the information transmission capacity, can be obtained. Hence, it is essential to design a special fiber supporting multiple OAM modes to shrink the time, enlarge the capacity, and improve the efficiency of data transmission. Photonic crystal fiber (PCF), a novel fiber for stable OAM modes transmission, has tremendous structural flexibility when compared with conventional fibers. The ideal performance parameters could beacquired by regulating the size and arrangement of holes in the microstructure fiber [11,12]. Furthermore, the distinctive fiber properties including endlessly single mode [13], tailorable dispersion [14], and confinement loss (CL) [13] influence the design of OAM fibers. The pioneering work of OAM transmission in PCF can be traced to Yue Y et al., who proposed a special PCF supporting the propagation of only two OAM modes [15]. Furthermore, the dispersion of the PCF is so large that it is impossible to consider it for practical applications. Gregg P et al. proposed an air-core fiber that supported 12 OAM states and experimentally demonstrated the stability of transmission of OAM modes in the fiber [16]. However, the PCF does not meet the demand for high-capacity communication. In 2017, Li HS et al. ingeniously proposed a Kagome hollow-core fiber, which is groundbreaking in terms of its geometry structure and transmitting frequency of the light incident on the fiber [18]. Nevertheless, the average CL of the vector modes is large (0.3dB/cm). Thus, it is considered impossible to apply this special OAM fiber to long-haul fiber communication systems. Moreover, a hollow-core PCF made of siliconwas reported for the first time in [19]. It proves the fact that crystalline silicon is a viable material for PCF. A major goal of this report is to enhance the communication quality and capacity. Accordingly, we propose a four-ring gradient-air-hole hollow ring-core PCF based on silicon and numerically simulate the novel PCF by using the full-vector finite element method (FEM) and perfectly matched layer (PML). In the designed PCF, the effective refractive index separation (Δn_eff_) between the vector modes (HE modes and EH modes) that could couple into the LP modes are larger than 1×10^−4^_;_ therefore, Δn_eff_ is large enough to hold 30 OAM states stably while propagating from the wavelength of 1.2 to 2.4 μm; thus, the data transmission capacity is greater than in [20]. Additionally, the CLs of most vector modes guided in this structure are lower by about 2 orders of magnitude than in [17] and the bandwidth of the OAM modes is wider than that of ordinary ring fibers [16,21–23]. The layout of this paper is as follows. First, the geometrical structure and the structural parameters of the designed PCF are introduced. The details of the supported OAM modes and several key performance parameters such as Δn_eff_, OAM phase, confinement loss, and dispersion of the proposed fiber are systematically investigated in a later section. In the next section, the comparisons between three four–ring gradient-air-hole silicon PCFs with different hole spacing are demonstrated. Finally, the conclusion and potential applications for this PCF are revealed.

## Materials and methods

Silicon was selected as the background material, for its high refractive index (n = 3.4764 at the wavelength of 1.55 μm) that is even larger than that of As_2_S_3_ (n = 2.4373 at the wavelength of 1.55 μm), which leads to a high material refractive index contrast. It is worthwhile to note that the high material refractive index contrast is beneficial for multimode propagation and prevents modal coupling.

As mentioned earlier, the proposed a four-ring gradient-air-hole hollow ring-core PCF based on silicon was numerically simulated by using the FEM and PML with COMSOL Multiphysics 5.2. As a professional simulation tool in the field of electromagnetics, COMSOL is superior to other PCF simulation tools. The FEM, which greatly improves the calculation precision and reduces the computation time, is advocated by COMSOL. Although there are many scientific methods for numerical calculation of the PCF, such as plane wave method, finite difference time domain, and supercell lattice method, FEM is the best candidate for the proposed PCF in this paper because FEM is suitable for a PCF that possesses air holes of different sizes. Furthermore, the PML is used as an absorbing boundary condition and it is efficacious in preventing the electromagnetic field distortion of the inner space of the PCF.

## Results and discussion

**Fig 1:**
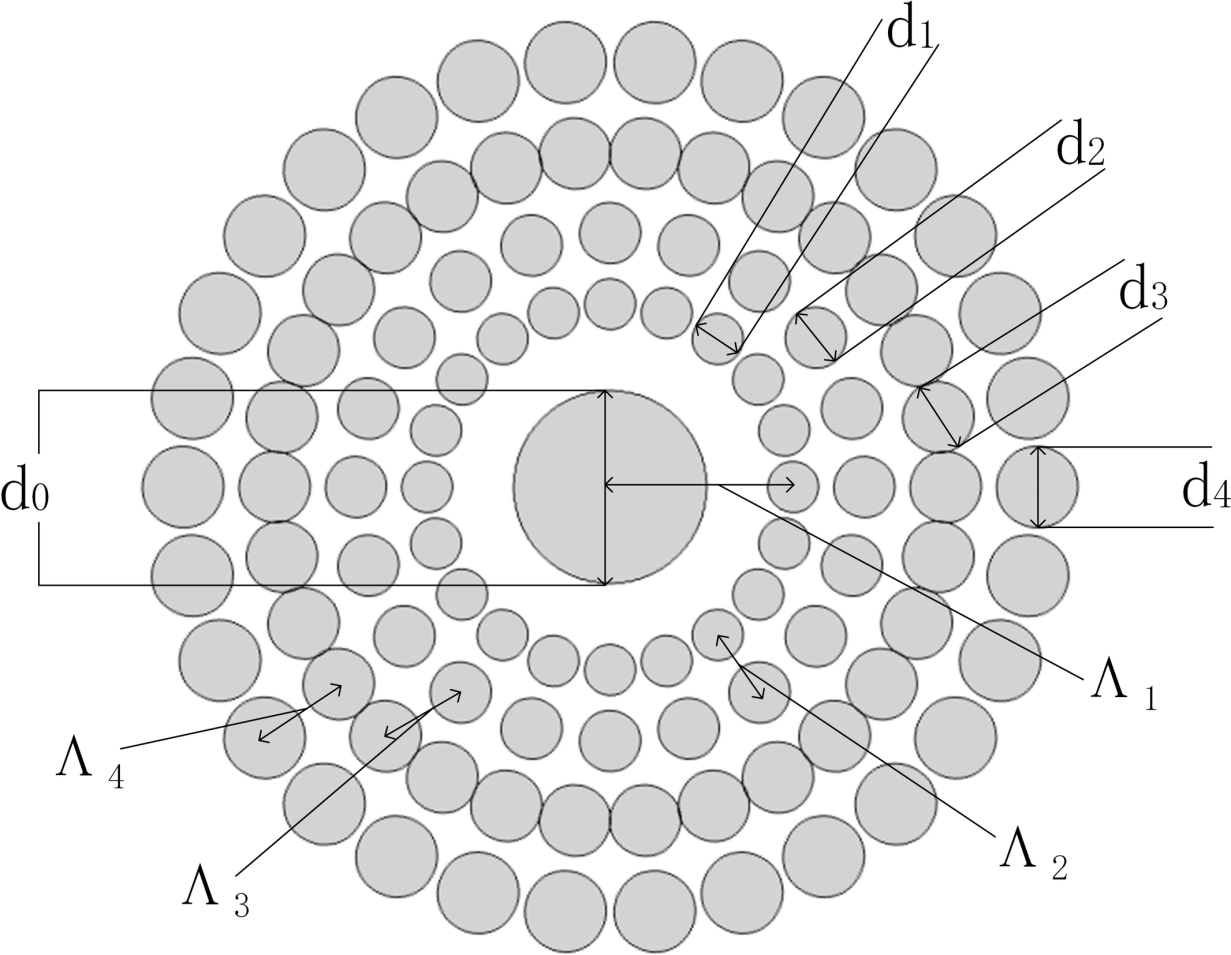
Cross-section of the designed PCF2#.

The cross-section of the proposed PCF2# is shown in Fig 1. The PCF comprises four rings containing air holes whose sizes are distributed in a gradient and the largest hole is at the center. The structural parameters of PCF2# are depicted in Table 1, where d_0_ is the diameter of the central hole, d_1_ to d_4_ are the diameters of the holes located in the different rings, and Λ_2_ to Λ_4_ are the hole pitches between the adjacent air holes of different rings. In this simulation, the number of conventional vector modes is sizable. At the same time, Δn_eff_ is appropriate for the OAM states. Consequently, 30 OAM modes can be obtained by coherently combining the even and odd hybrid modes of HE or EH. We can calculate the OAM states by the formulas [23]

$$\begin{array}{c} OAM_{\pm l,m}^{\pm }=HE_{l+1,m}^{even}\pm jHE_{l+1,m}^{odd},\\ OAM_{\pm l,m}^{\mp }=EH_{l-1,m}^{even}\pm jEH_{l-1,m}^{odd}, \end{array}$$ (1)

where the superscript in the OAM modes represents the direction of the circular polarization corresponding to the spin angular momentum and the subscript represents the topological charges, which reveal the direction of the helical phase wavefont rotation. The signs of the topological charges change based on the direction of circular polarization regardless of the HE modes or the EH modes. Herein, we explore the quantity and types of the OAM states. The conventional vector modes guided in this PCF are HE_91_-HE_11_, EH_71_-EH_11_, TE_01_, and TM_01_. Using the two formulas in Eq (1), we can obtain the following OAM modes: OAM± ±8,1 (EH_71_, HE_91_), OAM± ±7,1 (EH_61_, HE_81_), OAM± ±6,1 (EH_51_, HE_71_), OAM± ±5,1 (EH_41_, HE_61_), OAM± ±4,1 (EH_31_, HE_51_), OAM± ±3,1 (EH_21_, HE_41_), OAM± ±2,1 (EH_11_, HE_31_), and OAM± ±1,1 (HE_21_). It should be pointed out that the designed PCF can support 30 OAM states with the bandwidth of 900 nm from 1.5 μm to 2.4 μm.

**Table 1:** Structural parameters of PCF.

Parameters	PCF1#	PCF2#	PCF3#
Λ_1_(μm)	**3.6**	**3.6**	**3.6**
Λ_2_	**1.3**	**1.4**	**1.5**
Λ_3_	**1.5**	**1.6**	**1.7**
Λ_4_	**1.7**	**1.8**	**1.9**
d_0_	**3.8**	**3.8**	**3.8**
d_1_(μm)	**1**	**1**	**1**
d_2_	**1.2**	**1.2**	**1.2**
d_3_	**1.4**	**1.4**	**1.4**
d_4_	**1.6**	**1.6**	**1.6**

**Fig 2:**
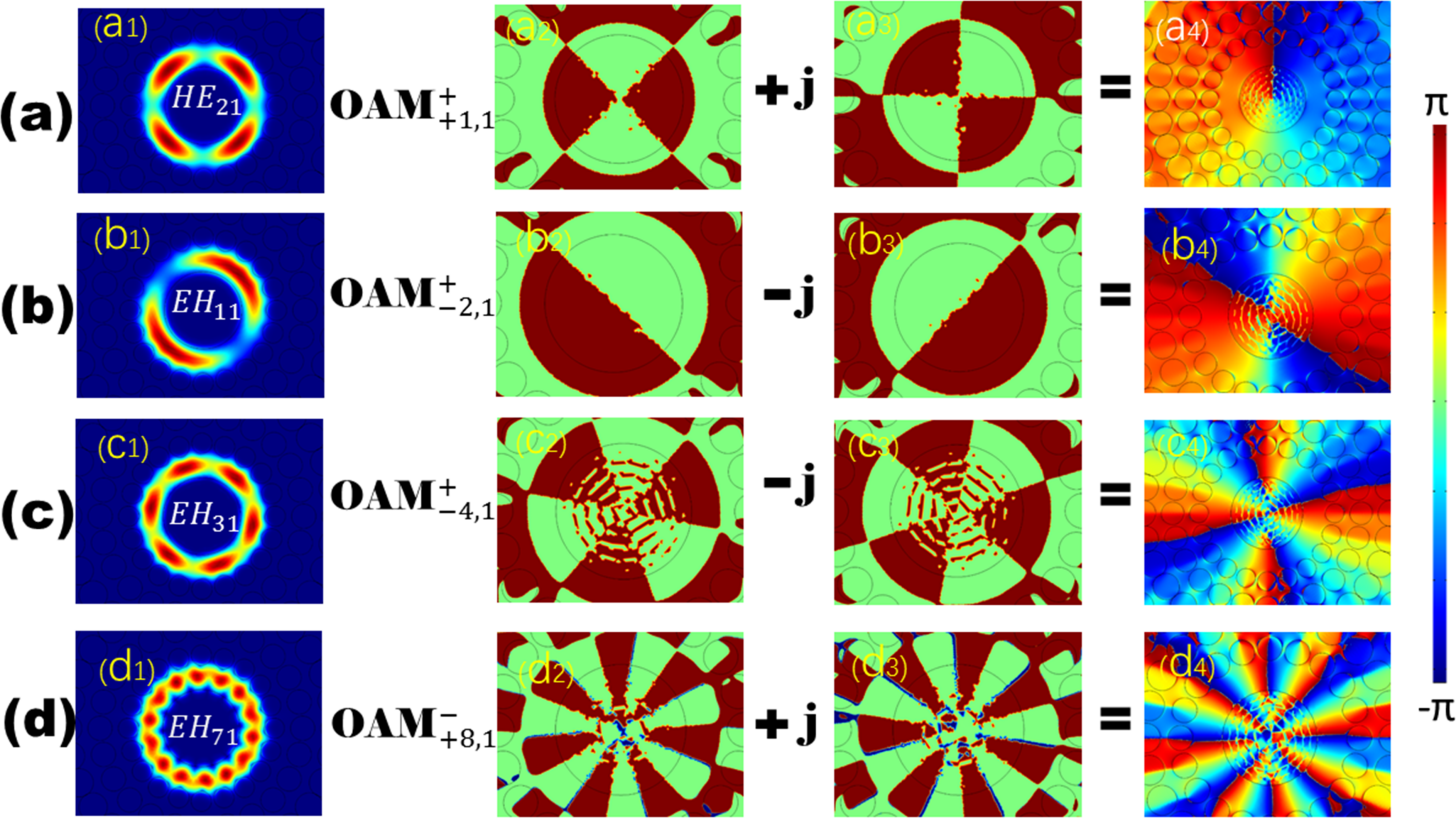
The first row contains the normalized intensity profiles of HE_21_, EH_11_, EH_31_, and EH_71_ modes, and the remaining rows show the generation progress of the phase distributions of the corresponding OAM modes in (a), (b), (c), and (d) respectively.

**Fig 3:**
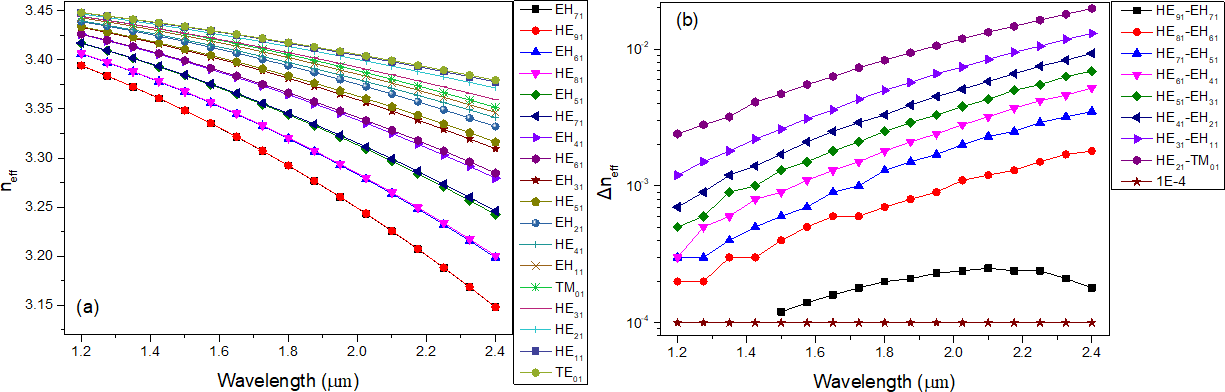
(a) n_eff_ of vector modes guided in PCF2# as a function of wavelength. (b) Δn_eff_ of vector modes guided in PCF2# as a function of wavelength.

The bandwidth of the designed 1# is much higher than that of conventional fibers [22]. Several sets of the electric field intensity profiles of the EH and HE modes and the corresponding OAM phase distributions are demonstrated in Fig 2, which indicate that this designed PCF is feasible for stable and multiple OAM mode transmission. The effective indices (n_eff_) of all the vector modes as a function of the wavelength ranging from the wavelength of 1.2 μm to 2.4 μm are shown in Fig 3(A). As seen from the curve, n_eff_ decreases with increasing wavelength and the HE_a1_ modes have larger n_eff_ compared with EH_a1_ (a = 1–7, representing the radial order of the modes). Fig 3(B) displays Δn_eff_ between HE_l+1,m_ and EH_l-1,m_ as well as between HE_21_ and TE_01_, TM_01._ We can see that all values of Δn_eff_ are above 1×10^−4^, which reveals the impossibility of the near-degenerate eigenmodes from coupling into the LP modes. As we know, Δn_eff_ must exceed 1×10^−4^ to prevent the near-degenerate vector eigenmodes from coupling into the LP modes [24]. Furthermore, half the values of Δn_eff_ between the modes are larger than 1×10^−3^ at the wavelength of 1.55 μm, where the modal crosstalk between the OAM states is maintained within an extremely small order of magnitude. In practice, the number of modes guided by the fiber also affects Δn_eff_ because all values of n_eff_ of the guided modes should lie between the refractive index of the cladding and that of the core. Δn_eff_ tends to become smaller with increase in the number of guide modes. Therefore, we encounter a trade-off between the number of vector modes guided by the PCF and the suitable Δn_eff_ between those modes while devising a special PCF. CL is a vital performance parameter, which plays decisive role in determing the transmission distance. Ultra-long haul optical fiber communication can be realized using the proposed PCF with an ultra-low CL and dispersion. The CL is currently a crucial factor that limits remote conveying. The CL of each mode guided in this PCF can be calculated by the following formula [25]:

L=2πλ20ln(10)106Im(neff)(dB/m), (2)

**Fig 4:**
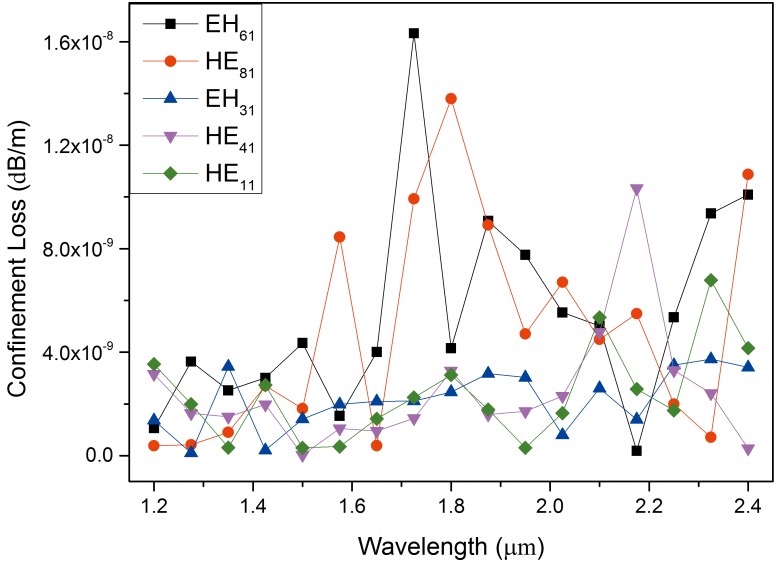
Confinement losses of the typical vector modes in PCF2# as a function of wavelength.

Where λ is the wavelength of the incident light, and Im(n_eff_) is the imaginary part of the modal effective indices. The CLs of the partial guided modes are shown in Fig 4. It can be seen that the average CL of these modes is approximately 1×10^−9^ dB/m, which is lower by about 3 orders of magnitude than the result in [24]. Especially, the CL of HE_11_ is near zero at the wavelength of 1.55 μm. The highest CL of the various modes with the bandwidth of 900 nm from 1.2 μm to 2.4 μm is approximately 1.6×10^−8^ dB/m. Hence, this special PCF could find potential application in long-distance optical fiber communication. In this study, the modal waveguide dispersion and material dispersion were systematically researched due to their effects on the light pulse.

Moreover, waveguide dispersion and material dispersion are the dominant types of dispersions, especially in PCF. It is meaningful to conduct an investigation on them. In this work, we calculated the gross dispersion of the vector modes by linearly combining the waveguide dispersion D_w_ and material dispersion D_m_. Herein, D_w_ and D_m_ are computed separately through the following formulas [25]:

$$D_{w}=\text{-}\frac{\lambda }{c}\frac{d^{2}n_{eff}}{d\lambda^{2}},$$ (3)

$$D_{m}=\text{-}\frac{\lambda }{c}\frac{d^{2}n\left(\lambda \right)}{d\lambda^{2}},$$ (4)

$$D\approx D_{w}+D_{m},$$ (5)

Where λ is the wavelength of the incident light, c is the light speed in vacuum, and n_eff_ is the effective indices of the vector mode.

**Fig 5:**
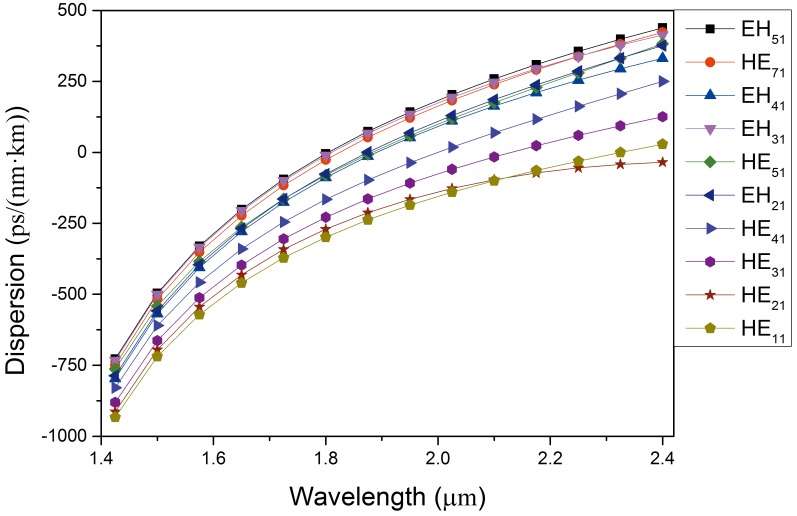
Dispersion curve of the guided modes in PCF2# as a function of wavelength.

**Fig 6:**
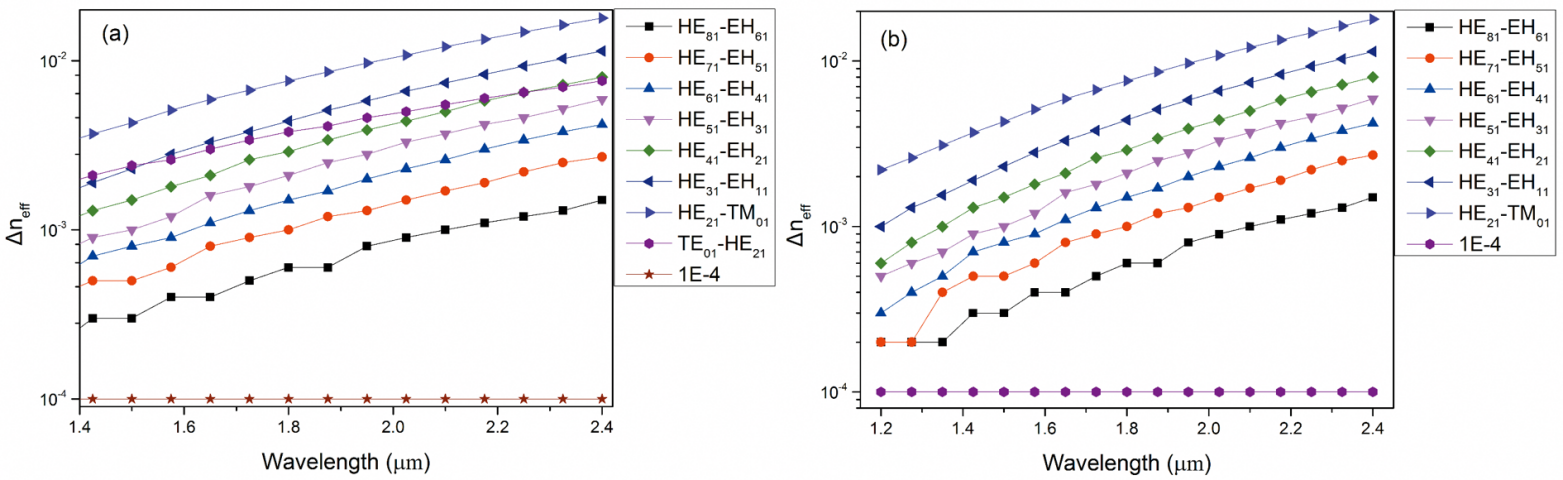
(a) Δn_eff_ of vector modes guided in PCF1# as a function of wavelength. (b) Δn_eff_ of vector modes guided in PCF3# as a function of wavelength.

Additionally, the material dispersion is determined by using Eq (4) along with the Sellmeier dispersion equation [26] as follows:

$$n^{2}=11.6858+\frac{0.939816}{\lambda^{2}}+\frac{0.00993358}{\lambda^{2}-1.22567},$$ (6)

where n is the refractive index of silicon and λ is the wavelength of the incident light. The gross dispersion is presented in Fig 5. It is apparent that the values of dispersion increase at a progressively slower rate. In addition, the modes guided in this PCF possess a bigger value of dispersion compared with conventional PCF, as silicon is selected as the background material. We discovered that a flatter dispersion curve can be obtained by reducing the value of d_0_; however, the number of OAM states guided in this PCF decreases with the decrease in d_0_. Given the trade-off between the quantity of OAM transmitted and the dispersion in this PCF, the trends observed in the above structural parameters are deemed reasonable. In fact, regulating the size, arrangement, or hole spacing between the air holes in this kind of PCF will affect the performance parameter. Here, three four-ring silicon PCFs with different hole spacing are comparatively researched. The structural parameters of three different PCFs are shown in Table 1. We mainly explored the quantity of vector modes (namely, the quantity of OAM modes) and dispersion performance in PCF1#, PCF2#, and PCF3#. The Δn_eff_ of PCF1# and PCF3# are presented in Fig 6(A) and 6(B), respectively. From Fig 6(A) and 6(B), we can find that the quantity of OAM states transmitted in PCF1# and PCF3# are 28, which is less than that in PCF2#. In addition, the bandwidth of the OAM modes covers only 375 nm (1.2–1.575 μm) in PCF3#.

**Fig 7:**
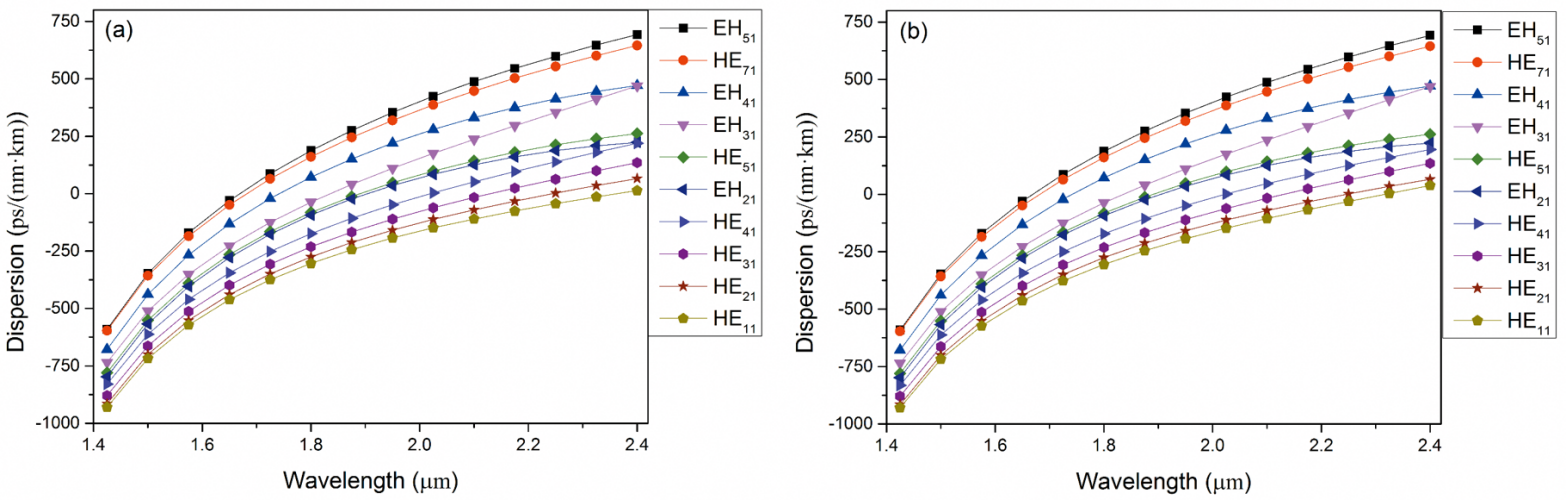
(a) Dispersion curve of guided modes in PCF1# as a function of wavelength. (b) Dispersion curve of guided modes in PCF3# as a function of wavelength.

Fig 7(A) and 7(B) reveals that PCF1# and PCF3# possess larger values of dispersion than PCF2# though the slopes of the dispersion curves are nearly equivalent. It is significant to note that dispersion in the PCF is allowed, but the smaller the dispersion, the better is the quality of optical fiber communications. By comparing the above three silicon PCFs, it can be concluded that PCF2# possesses better communication performance than PCF1# and PCF3#.

PCF possesses many unique characteristics such as endlessly single mode, tailorable dispersion, and low confinement loss. Compared with a traditional fiber, it shows a greater capacity for information transmission to further promote the transmission efficiency. In addition, PCF has more structural flexibility when compared with conventional fibers. It may be applied in the field of sensing, communications, or laser. However, its subtle structure causes increased fabrication complexity and cost when compared with traditional fiber. The most critical challenge with regard to fabrication is maintaining the air holes at the stated size and arrangement, as the holes are close to each other and are tiny. Nevertheless, we believe that the proposed PCF could be realized with the development of manufacturing technology in the future.

## Conclusion

In summary, we have proposed a novel four-ring hollow-core silicon PCF2# and presented a systematical investigation of the properties of the vector modes in this paper. The PCF can stably support 30 OAM states from the wavelength of 1.5 μm to 2.4 μm with a large Δn_eff_ of above 1×10^−4^. We notice that the confinement loss is less than 1×10^−9^ dB/m at the wavelength of 1.55 μm and the average confinement loss is less than 1×10^−8^ dB/m from the wavelength of 1.2 μm to 2.4 μm. In addition, the dispersion values are larger than that of the conventional ring-core fiber because of the use of crystalline silicon as the background material of the PCF. In addition, we compared three four-ring hollow-core silicon PCFs and aconcluded that PCF2# possesses better communication performance than PCF1# and PCF3#. In summary, this fiber structure will be a potential candidate for high-capacity optical fiber communications and OAM sensing applications using fibers.

## Supporting information

S1 Dataset The original datasets containing the effective refractive indices of all vector modes in PCF2#. (DOCX)

S1 Table The original dataset containing the real parts of the effective refractive indices of all vector modes in PCF2#. (PDF)

S2 Table The original dataset containing the real parts of the effective refractive index separation between HE_l+1,m_ and EH_l-1,m_ modes in PCF2#. (PDF)

S3 Table The original dataset containing the imaginary parts of the effective refractive indices of all vector modes in PCF2#. (PDF)

S4 Table The original dataset containing the confinement losses of all vector modes in PCF2#. (PDF)

S5 Table The original dataset containing the dispersion of all vector modes in PCF2#. (PDF)

S6 Table The original dataset containing the real part of the effective refractive index separation between HE_l+1,m_ and EH_l-1,m_ modes in PCF1#. (PDF)

S7 Table The original dataset containing the real part of the effective refractive index separation between HE_l+1,m_ and EH_l-1,m_ modes in PCF3#. (PDF)

S8 Table The original dataset containing the dispersion of all vector modes in PCF1#. (PDF)

S9 Table The original dataset containing the dispersion of all vector modes in PCF3#. (PDF)

S1 File The COMSOL batch file of the proposed PCF2#. (JAVA)

S2 File The COMSOL batch file of PCF1#. (JAVA)

S3 File The COMSOL batch file of PCF3#. (JAVA)
