# Supplementary material for: Theoretical proposal of a low-loss wide-bandwidth silicon photonic crystal fiber for supporting 30 orbital angular momentum modes
Source: PLoS One. 2017 Dec 13;12(12):e0189660. doi: 10.1371/journal.pone.0189660 (PMC5728573; doi:10.1371/journal.pone.0189660)
Supplement: S1 Dataset — (DOCX) [file pone.0189660.s001.docx]

1.2000 3.4171-1.1080E-4i

1.2000 3.4174-1.1003E-4i

1.2000 3.4163-1.0405E-4i

1.2000 3.4171-9.9746E-5i

1.2000 3.4150-9.4545E-5i

1.2000 3.4374-9.4509E-5i

1.2000 3.4382-8.9597E-5i

1.2000 3.4380-8.8498E-5i

1.2000 3.4134-8.4978E-5i

1.2000 3.4163-8.4305E-5i

1.2000 3.4382-8.4023E-5i

1.2000 3.4378-8.2434E-5i

1.2000 3.4364-8.0893E-5i

1.2000 3.4370-7.8926E-5i

1.2000 3.4378-7.8640E-5i

1.2000 3.4351-7.7395E-5i

1.2000 3.4382-7.6870E-5i

1.2000 3.4358-7.5656E-5i

1.2000 3.4380-7.5234E-5i

1.2000 3.4370-7.2080E-5i

1.2000 3.4150-6.6956E-5i

1.2000 3.4328-6.6007E-5i

1.2000 3.4336-6.5695E-5i

1.2000 3.4116-6.5231E-5i

1.2000 3.4343-6.4121E-5i

1.2000 3.4364-6.4214E-5i

1.2000 3.4358-6.2604E-5i

1.2000 3.4374-6.2101E-5i

1.2000 3.4321-6.1900E-5i

1.2000 3.4312-6.0868E-5i

1.2000 3.4315-5.9308E-5i

1.2000 3.4343-5.8985E-5i

1.2000 3.4351-5.3466E-5i

1.2000 3.4311-5.1948E-5i

1.2000 3.4335-5.0974E-5i

1.2000 3.4134-4.9696E-5i

1.2000 3.4315-4.9398E-5i

1.2000 3.4321-4.7400E-5i

1.2000 3.4095-4.5844E-5i

1.2000 3.4328-4.5750E-5i

1.2000 3.4312-4.5414E-5i

1.2000 3.4116-4.4945E-5i

1.2000 3.4072-4.2343E-5i

1.2000 3.4095-4.2233E-5i

1.2000 3.4048-4.1298E-5i

1.2000 3.4072-2.6078E-5i

1.2000 3.4023-1.9665E-5i

1.2000 3.3996-1.5223E-5i

1.2000 3.4023-1.4573E-5i

1.2000 3.3968-9.6589E-6i

1.2000 3.4048-9.2368E-6i

1.2000 3.3996-4.0611E-6i

1.2000 3.3939-3.1616E-6i

1.2000 3.3942+9.1753E-17i

1.2000 3.3942+5.9996E-17i

1.2000 3.3943+7.3417E-17i

1.2000 3.3943+1.0063E-16i

1.2000 3.4063-2.3413E-17i

1.2000 3.4063-3.9001E-17i

1.2000 3.4065-8.3911E-18i

1.2000 3.4065+1.6529E-17i

1.2000 3.4169-3.1904E-17i

1.2000 3.4169-6.9477E-17i

1.2000 3.4172-2.1889E-18i

1.2000 3.4172+1.5560E-17i

1.2000 3.4260-5.4562E-17i

1.2000 3.4260+8.8475E-17i

1.2000 3.4263+7.2589E-17i

1.2000 3.4264-8.6914E-17i

1.2000 3.4334+2.9965E-17i

1.2000 3.4334+1.9760E-17i

1.2000 3.4339-2.8203E-17i

1.2000 3.4339+6.5250E-18i

1.2000 3.4391-2.3135E-17i

1.2000 3.4391-1.3610E-17i

1.2000 3.4398-6.8369E-17i

1.2000 3.4398-6.9256E-17i

1.2000 3.4429+2.4372E-17i

1.2000 3.4429-1.9289E-17i

1.2000 3.4441+4.1146E-18i

1.2000 3.4441-2.1358E-17i

1.2000 3.4444-1.0414E-16i

1.2000 3.4468-3.0448E-18i

1.2000 3.4468-2.7107E-17i

1.2000 3.4480-7.7862E-17i

1.2000 3.4480-7.5488E-17i

1.2000 3.4482-2.2018E-17i

1.2000 3.3968+3.9681E-6i

1.2000 3.3939+9.2264E-6i

1.2000 3.3918+3.6410E-4i

1.2000 3.4348+3.7248E-4i

1.2000 3.4354+3.7882E-4i

1.2000 3.4345+3.9143E-4i

1.2000 3.4362+3.9244E-4i

1.2000 3.4371+4.0114E-4i

1.2000 3.4347+4.1304E-4i

1.2000 3.4381+4.1665E-4i

1.2000 3.4397+4.1890E-4i

1.2000 3.4354+4.1991E-4i

1.2000 3.4421+4.2425E-4i

1.2000 3.4362+4.2838E-4i

1.2000 3.4411+4.3412E-4i

1.2000 3.4404+4.3437E-4i

1.2000 3.4389+4.3822E-4i

1.2000 3.4371+4.4211E-4i

1.2000 3.4389+4.4740E-4i

1.2000 3.4380+4.4817E-4i

1.2000 3.4429+4.5322E-4i

1.2000 3.4428+4.5365E-4i

1.2000 3.4424+4.5692E-4i

1.2000 3.4416+4.5710E-4i

1.2000 3.4428+4.5807E-4i

1.2000 3.4426+4.6804E-4i

1.2000 3.4424+4.6904E-4i

1.2000 3.3990+4.7145E-4i

1.2000 3.4404+4.7255E-4i

1.2000 3.3961+4.7307E-4i

1.2000 3.4416+4.7438E-4i

1.2000 3.4397+4.7608E-4i

1.2000 3.4019+4.7670E-4i

1.2000 3.4125+4.7741E-4i

1.2000 3.4047+4.7947E-4i

1.2000 3.4426+4.8115E-4i

1.2000 3.4410+4.8298E-4i

1.2000 3.4075+4.8500E-4i

1.2000 3.4019+4.9147E-4i

1.2000 3.3990+4.9301E-4i

1.2000 3.4047+4.9678E-4i

1.2000 3.4100+4.9781E-4i

1.2000 3.3960+5.0040E-4i

1.2000 3.4074+5.0217E-4i

1.2000 3.4148+5.0250E-4i

1.2000 3.4100+5.0353E-4i

1.2000 3.4420+5.0437E-4i

1.2000 3.4191+5.1685E-4i

1.2000 3.4170+5.2290E-4i

1.2000 3.3938+5.3101E-4i

1.2000 3.4209+5.3261E-4i

1.2000 3.4148+5.3320E-4i

1.2000 3.4170+5.3825E-4i

1.2000 3.4124+5.3998E-4i

1.2000 3.4225+5.4874E-4i

1.2000 3.4190+5.7481E-4i

1.2000 3.4237+5.8576E-4i

1.2000 3.4208+5.9519E-4i

1.2000 3.4245+6.1480E-4i

1.2000 3.4224+6.2133E-4i

1.2000 3.4247+6.2409E-4i

1.2000 3.4236+6.3313E-4i

1.2000 3.4244+6.4090E-4i

1.2750 3.4095-1.3362E-4i

1.2750 3.4098-1.3218E-4i

1.2750 3.4086-1.2687E-4i

1.2750 3.4095-1.2045E-4i

1.2750 3.4071-1.1664E-4i

1.2750 3.4325-1.1406E-4i

1.2750 3.4333-1.0766E-4i

1.2750 3.4053-1.0694E-4i

1.2750 3.4332-1.0641E-4i

1.2750 3.4086-1.0322E-4i

1.2750 3.4334-1.0097E-4i

1.2750 3.4329-9.9332E-5i

1.2750 3.4314-9.8304E-5i

1.2750 3.4320-9.5606E-5i

1.2750 3.4299-9.4711E-5i

1.2750 3.4329-9.4780E-5i

1.2750 3.4306-9.2288E-5i

1.2750 3.4333-9.2359E-5i

1.2750 3.4332-9.0396E-5i

1.2750 3.4320-8.7454E-5i

1.2750 3.4032-8.4123E-5i

1.2750 3.4071-8.4071E-5i

1.2750 3.4272-8.1573E-5i

1.2750 3.4281-8.1227E-5i

1.2750 3.4290-7.9156E-5i

1.2750 3.4313-7.8331E-5i

1.2750 3.4306-7.6837E-5i

1.2750 3.4264-7.6538E-5i

1.2750 3.4325-7.5157E-5i

1.2750 3.4254-7.3691E-5i

1.2750 3.4258-7.2962E-5i

1.2750 3.4290-7.3058E-5i

1.2750 3.4298-6.6152E-5i

1.2750 3.4053-6.4646E-5i

1.2750 3.4281-6.3707E-5i

1.2750 3.4009-6.2038E-5i

1.2750 3.4253-6.2144E-5i

1.2750 3.4258-6.1141E-5i

1.2750 3.4032-6.0322E-5i

1.2750 3.4264-5.9395E-5i

1.2750 3.3984-5.8526E-5i

1.2750 3.4009-5.7809E-5i

1.2750 3.3956-5.7645E-5i

1.2750 3.4272-5.7617E-5i

1.2750 3.4254-5.5171E-5i

1.2750 3.3983-3.9210E-5i

1.2750 3.3927-3.2468E-5i

1.2750 3.3897-2.7605E-5i

1.2750 3.3927-2.6438E-5i

1.2750 3.3866-2.1539E-5i

1.2750 3.3956-1.9693E-5i

1.2750 3.3897-1.4465E-5i

1.2750 3.3833-1.4117E-5i

1.2750 3.3866-5.1662E-6i

1.2750 3.3837+6.0327E-17i

1.2750 3.3837+1.1731E-16i

1.2750 3.3838-3.2904E-17i

1.2750 3.3838-8.1042E-17i

1.2750 3.3974-8.4908E-17i

1.2750 3.3974+5.8635E-17i

1.2750 3.3976-9.8843E-18i

1.2750 3.3976-5.7479E-17i

1.2750 3.4094+4.0140E-18i

1.2750 3.4094+2.0032E-17i

1.2750 3.4097-1.0816E-17i

1.2750 3.4097+1.5750E-17i

1.2750 3.4196-7.5150E-17i

1.2750 3.4196-6.4908E-17i

1.2750 3.4201-3.3338E-17i

1.2750 3.4201-7.1728E-17i

1.2750 3.4280-2.3830E-18i

1.2750 3.4280-2.7769E-17i

1.2750 3.4286-3.7814E-17i

1.2750 3.4286-5.1967E-17i

1.2750 3.4344+2.8908E-17i

1.2750 3.4344+4.0218E-17i

1.2750 3.4353+1.6129E-17i

1.2750 3.4353+3.8265E-17i

1.2750 3.4387+1.8476E-17i

1.2750 3.4387+2.3670E-17i

1.2750 3.4402+8.3798E-18i

1.2750 3.4402-4.8567E-17i

1.2750 3.4404+7.1010E-17i

1.2750 3.4432+2.4767E-17i

1.2750 3.4432-2.8867E-17i

1.2750 3.4446+4.6528E-17i

1.2750 3.4446+2.8431E-17i

1.2750 3.4448-6.4797E-18i

1.2750 3.3833+7.5785E-7i

1.2750 3.4297+4.3884E-4i

1.2750 3.3810+4.4670E-4i

1.2750 3.4304+4.4762E-4i

1.2750 3.4294+4.6128E-4i

1.2750 3.4314+4.6397E-4i

1.2750 3.4324+4.7442E-4i

1.2750 3.4296+4.8766E-4i

1.2750 3.4335+4.9206E-4i

1.2750 3.4354+4.9364E-4i

1.2750 3.4304+4.9632E-4i

1.2750 3.4381+4.9841E-4i

1.2750 3.4313+5.0713E-4i

1.2750 3.4369+5.1123E-4i

1.2750 3.4362+5.1209E-4i

1.2750 3.4344+5.1793E-4i

1.2750 3.4324+5.2333E-4i

1.2750 3.4344+5.2891E-4i

1.2750 3.4334+5.3073E-4i

1.2750 3.4389+5.3191E-4i

1.2750 3.4389+5.3287E-4i

1.2750 3.4384+5.3708E-4i

1.2750 3.4388+5.3717E-4i

1.2750 3.4375+5.3848E-4i

1.2750 3.4384+5.5179E-4i

1.2750 3.4387+5.5253E-4i

1.2750 3.4361+5.5765E-4i

1.2750 3.4375+5.5894E-4i

1.2750 3.4353+5.6302E-4i

1.2750 3.3893+5.6350E-4i

1.2750 3.4387+5.6429E-4i

1.2750 3.3860+5.6513E-4i

1.2750 3.4046+5.6697E-4i

1.2750 3.3926+5.6932E-4i

1.2750 3.4368+5.6971E-4i

1.2750 3.3958+5.7154E-4i

1.2750 3.3989+5.7775E-4i

1.2750 3.3926+5.8697E-4i

1.2750 3.3893+5.8948E-4i

1.2750 3.4018+5.9231E-4i

1.2750 3.3958+5.9323E-4i

1.2750 3.4379+5.9444E-4i

1.2750 3.4073+5.9561E-4i

1.2750 3.3859+5.9899E-4i

1.2750 3.3988+5.9897E-4i

1.2750 3.4018+5.9998E-4i

1.2750 3.4121+6.1070E-4i

1.2750 3.4097+6.1982E-4i

1.2750 3.4142+6.2854E-4i

1.2750 3.3835+6.3044E-4i

1.2750 3.4072+6.3368E-4i

1.2750 3.4097+6.3791E-4i

1.2750 3.4045+6.4277E-4i

1.2750 3.4160+6.4586E-4i

1.2750 3.4120+6.8101E-4i

1.2750 3.4173+6.8850E-4i

1.2750 3.4140+7.0343E-4i

1.2750 3.4183+7.2158E-4i

1.2750 3.4186+7.3214E-4i

1.2750 3.4158+7.3343E-4i

1.2750 3.4173+7.4567E-4i

1.2750 3.4182+7.5326E-4i

1.3500 3.4014-1.5936E-4i

1.3500 3.4018-1.5716E-4i

1.3500 3.4004-1.5273E-4i

1.3500 3.4014-1.4382E-4i

1.3500 3.3988-1.4181E-4i

1.3500 3.4272-1.3613E-4i

1.3500 3.3968-1.3204E-4i

1.3500 3.4282-1.2803E-4i

1.3500 3.4280-1.2662E-4i

1.3500 3.4004-1.2465E-4i

1.3500 3.4283-1.2008E-4i

1.3500 3.4277-1.1837E-4i

1.3500 3.4260-1.1799E-4i

1.3500 3.4267-1.1444E-4i

1.3500 3.4243-1.1432E-4i

1.3500 3.4277-1.1296E-4i

1.3500 3.4252-1.1109E-4i

1.3500 3.4282-1.0982E-4i

1.3500 3.4280-1.0749E-4i

1.3500 3.3944-1.0587E-4i

1.3500 3.4267-1.0482E-4i

1.3500 3.3988-1.0361E-4i

1.3500 3.4214-9.9268E-5i

1.3500 3.4224-9.8888E-5i

1.3500 3.4234-9.6244E-5i

1.3500 3.4260-9.4306E-5i

1.3500 3.4205-9.3210E-5i

1.3500 3.4252-9.2992E-5i

1.3500 3.4272-8.9894E-5i

1.3500 3.4234-8.9059E-5i

1.3500 3.4197-8.8541E-5i

1.3500 3.4193-8.8191E-5i

1.3500 3.3968-8.1855E-5i

1.3500 3.3918-8.0926E-5i

1.3500 3.4243-8.0515E-5i

1.3500 3.3944-7.8211E-5i

1.3500 3.4224-7.8227E-5i

1.3500 3.3889-7.7537E-5i

1.3500 3.3859-7.6974E-5i

1.3500 3.3918-7.6007E-5i

1.3500 3.4197-7.4563E-5i

1.3500 3.4192-7.3528E-5i

1.3500 3.4205-7.3136E-5i

1.3500 3.4214-7.1202E-5i

1.3500 3.4193-6.6220E-5i

1.3500 3.3889-5.4830E-5i

1.3500 3.3826-4.7837E-5i

1.3500 3.3793-4.2538E-5i

1.3500 3.3826-4.0803E-5i

1.3500 3.3757-3.6044E-5i

1.3500 3.3859-3.2505E-5i

1.3500 3.3720-2.7776E-5i

1.3500 3.3792-2.7245E-5i

1.3500 3.3757-1.6614E-5i

1.3500 3.3720-1.0149E-5i

1.3500 3.3725-1.8109E-16i

1.3500 3.3725-2.5983E-16i

1.3500 3.3726-1.6428E-17i

1.3500 3.3726+1.2609E-16i

1.3500 3.3879+6.2453E-17i

1.3500 3.3879-4.9027E-17i

1.3500 3.3882+2.2403E-17i

1.3500 3.3882-2.5474E-17i

1.3500 3.4014+7.4604E-18i

1.3500 3.4014+1.4850E-17i

1.3500 3.4018+6.4908E-17i

1.3500 3.4018+5.9403E-17i

1.3500 3.4129+4.8909E-17i

1.3500 3.4129+8.1864E-17i

1.3500 3.4135+1.0968E-16i

1.3500 3.4135+2.4529E-16i

1.3500 3.4222+8.5108E-17i

1.3500 3.4222+6.0924E-17i

1.3500 3.4231+7.1564E-17i

1.3500 3.4231+5.2876E-17i

1.3500 3.4294+1.1527E-16i

1.3500 3.4294+2.4981E-17i

1.3500 3.4306-3.9544E-17i

1.3500 3.4306-3.7117E-17i

1.3500 3.4343-7.7195E-17i

1.3500 3.4343+2.6892E-17i

1.3500 3.4361-4.0547E-18i

1.3500 3.4361-4.6347E-17i

1.3500 3.4361+5.0309E-17i

1.3500 3.4395+2.2963E-17i

1.3500 3.4395-2.4882E-17i

1.3500 3.4411+7.8157E-18i

1.3500 3.4411+3.4355E-17i

1.3500 3.4414-1.5104E-17i

1.3500 3.4244+5.1169E-4i

1.3500 3.4252+5.2334E-4i

1.3500 3.4240+5.3807E-4i

1.3500 3.3697+5.4050E-4i

1.3500 3.4263+5.4270E-4i

1.3500 3.4275+5.5508E-4i

1.3500 3.4243+5.6976E-4i

1.3500 3.4286+5.7493E-4i

1.3500 3.4308+5.7560E-4i

1.3500 3.4338+5.7949E-4i

1.3500 3.4252+5.8044E-4i

1.3500 3.4262+5.9388E-4i

1.3500 3.4325+5.9575E-4i

1.3500 3.4317+5.9735E-4i

1.3500 3.4297+6.0559E-4i

1.3500 3.4274+6.1268E-4i

1.3500 3.4348+6.1794E-4i

1.3500 3.4297+6.1848E-4i

1.3500 3.4348+6.1953E-4i

1.3500 3.4286+6.2165E-4i

1.3500 3.4347+6.2353E-4i

1.3500 3.4342+6.2481E-4i

1.3500 3.4332+6.2771E-4i

1.3500 3.4342+6.4242E-4i

1.3500 3.4345+6.4582E-4i

1.3500 3.4317+6.5105E-4i

1.3500 3.4332+6.5170E-4i

1.3500 3.4345+6.5471E-4i

1.3500 3.4307+6.5862E-4i

1.3500 3.4325+6.6490E-4i

1.3500 3.3791+6.6559E-4i

1.3500 3.3963+6.6562E-4i

1.3500 3.3754+6.6721E-4i

1.3500 3.3828+6.7198E-4i

1.3500 3.3864+6.7346E-4i

1.3500 3.3899+6.8027E-4i

1.3500 3.3828+6.9282E-4i

1.3500 3.4337+6.9328E-4i

1.3500 3.3791+6.9646E-4i

1.3500 3.3931+6.9662E-4i

1.3500 3.3993+6.9804E-4i

1.3500 3.3863+7.0020E-4i

1.3500 3.3898+7.0610E-4i

1.3500 3.3931+7.0652E-4i

1.3500 3.3753+7.0844E-4i

1.3500 3.4047+7.1357E-4i

1.3500 3.4021+7.2642E-4i

1.3500 3.4071+7.3357E-4i

1.3500 3.3725+7.4044E-4i

1.3500 3.3992+7.4437E-4i

1.3500 3.4020+7.4735E-4i

1.3500 3.4091+7.5201E-4i

1.3500 3.3962+7.5617E-4i

1.3500 3.4046+7.9762E-4i

1.3500 3.4107+8.0079E-4i

1.3500 3.4069+8.2206E-4i

1.3500 3.4118+8.3811E-4i

1.3500 3.4121+8.5001E-4i

1.3500 3.4090+8.5623E-4i

1.3500 3.4106+8.6879E-4i

1.3500 3.4117+8.7608E-4i

1.4250 3.3929-1.8821E-4i

1.4250 3.3933-1.8515E-4i

1.4250 3.3917-1.8179E-4i

1.4250 3.3900-1.7031E-4i

1.4250 3.3929-1.7004E-4i

1.4250 3.4217-1.6089E-4i

1.4250 3.3877-1.6039E-4i

1.4250 3.4228-1.5085E-4i

1.4250 3.4226-1.4928E-4i

1.4250 3.3917-1.4873E-4i

1.4250 3.4229-1.4149E-4i

1.4250 3.4203-1.4011E-4i

1.4250 3.4222-1.3966E-4i

1.4250 3.4185-1.3636E-4i

1.4250 3.4211-1.3557E-4i

1.4250 3.4222-1.3330E-4i

1.4250 3.4195-1.3220E-4i

1.4250 3.3851-1.3063E-4i

1.4250 3.4228-1.2936E-4i

1.4250 3.4226-1.2663E-4i

1.4250 3.3899-1.2573E-4i

1.4250 3.4211-1.2431E-4i

1.4250 3.4152-1.1922E-4i

1.4250 3.4163-1.1881E-4i

1.4250 3.4174-1.1550E-4i

1.4250 3.4203-1.1225E-4i

1.4250 3.4142-1.1205E-4i

1.4250 3.4194-1.1120E-4i

1.4250 3.4174-1.0711E-4i

1.4250 3.4217-1.0641E-4i

1.4250 3.4134-1.0619E-4i

1.4250 3.4129-1.0448E-4i

1.4250 3.3821-1.0268E-4i

1.4250 3.3877-1.0143E-4i

1.4250 3.3756-9.9460E-5i

1.4250 3.3790-9.9538E-5i

1.4250 3.3851-9.8780E-5i

1.4250 3.3822-9.6990E-5i

1.4250 3.4185-9.6709E-5i

1.4250 3.4163-9.4643E-5i

1.4250 3.4134-8.9792E-5i

1.4250 3.4142-8.8746E-5i

1.4250 3.4152-8.6619E-5i

1.4250 3.4127-8.6129E-5i

1.4250 3.4129-7.8646E-5i

1.4250 3.3790-7.3072E-5i

1.4250 3.3720-6.6055E-5i

1.4250 3.3682-6.0243E-5i

1.4250 3.3720-5.7917E-5i

1.4250 3.3642-5.3310E-5i

1.4250 3.3756-4.7790E-5i

1.4250 3.3601-4.4322E-5i

1.4250 3.3682-4.2604E-5i

1.4250 3.3642-3.0545E-5i

1.4250 3.3601-2.3661E-5i

1.4250 3.3608-1.4771E-16i

1.4250 3.3608-7.3548E-17i

1.4250 3.3609-1.5593E-16i

1.4250 3.3609-2.0120E-16i

1.4250 3.3779+7.8546E-17i

1.4250 3.3779+7.4640E-17i

1.4250 3.3782+7.0421E-17i

1.4250 3.3782-8.6051E-17i

1.4250 3.3930-1.5877E-17i

1.4250 3.3930-4.4740E-17i

1.4250 3.3935-5.5321E-19i

1.4250 3.3935-9.5535E-18i

1.4250 3.4057+3.8513E-17i

1.4250 3.4057-6.0366E-19i

1.4250 3.4065-4.5893E-17i

1.4250 3.4065+1.7127E-16i

1.4250 3.4162-5.4467E-18i

1.4250 3.4162-3.9105E-18i

1.4250 3.4172+1.0803E-16i

1.4250 3.4172+4.1569E-17i

1.4250 3.4242+1.5857E-17i

1.4250 3.4242+1.5767E-17i

1.4250 3.4256-5.3535E-17i

1.4250 3.4256-5.1459E-17i

1.4250 3.4295+6.3416E-18i

1.4250 3.4295+1.0468E-16i

1.4250 3.4315+7.3510E-17i

1.4250 3.4317-1.0706E-17i

1.4250 3.4317+2.2939E-17i

1.4250 3.4356-2.5441E-17i

1.4250 3.4356+9.0912E-18i

1.4250 3.4374+7.1141E-17i

1.4250 3.4374+3.3017E-17i

1.4250 3.4378-3.4894E-17i

1.4250 3.4188+5.9122E-4i

1.4250 3.4197+6.0618E-4i

1.4250 3.4184+6.2193E-4i

1.4250 3.4210+6.2880E-4i

1.4250 3.4223+6.4325E-4i

1.4250 3.3577+6.4599E-4i

1.4250 3.4187+6.5954E-4i

1.4250 3.4261+6.6486E-4i

1.4250 3.4236+6.6541E-4i

1.4250 3.4295+6.6759E-4i

1.4250 3.4197+6.7244E-4i

1.4250 3.4280+6.8781E-4i

1.4250 3.4209+6.8882E-4i

1.4250 3.4271+6.9029E-4i

1.4250 3.4248+7.0135E-4i

1.4250 3.4222+7.1032E-4i

1.4250 3.4305+7.1138E-4i

1.4250 3.4305+7.1373E-4i

1.4250 3.4248+7.1627E-4i

1.4250 3.4304+7.1724E-4i

1.4250 3.4299+7.2022E-4i

1.4250 3.4235+7.2109E-4i

1.4250 3.4287+7.2491E-4i

1.4250 3.4299+7.4104E-4i

1.4250 3.4302+7.4687E-4i

1.4250 3.4287+7.5279E-4i

1.4250 3.4270+7.5289E-4i

1.4250 3.4302+7.5366E-4i

1.4250 3.4259+7.6305E-4i

1.4250 3.4279+7.6868E-4i

1.4250 3.3876+7.7344E-4i

1.4250 3.3683+7.7805E-4i

1.4250 3.3641+7.7957E-4i

1.4250 3.3725+7.8505E-4i

1.4250 3.3765+7.8549E-4i

1.4250 3.3803+7.9276E-4i

1.4250 3.4293+8.0103E-4i

1.4250 3.3724+8.0938E-4i

1.4250 3.3909+8.1003E-4i

1.4250 3.3840+8.1092E-4i

1.4250 3.3683+8.1427E-4i

1.4250 3.3764+8.1792E-4i

1.4250 3.3840+8.2337E-4i

1.4250 3.3803+8.2380E-4i

1.4250 3.3970+8.2558E-4i

1.4250 3.3640+8.2905E-4i

1.4250 3.3940+8.4287E-4i

1.4250 3.3997+8.4783E-4i

1.4250 3.3610+8.6128E-4i

1.4250 3.3908+8.6548E-4i

1.4250 3.3940+8.6670E-4i

1.4250 3.4020+8.6727E-4i

1.4250 3.3875+8.8036E-4i

1.4250 3.4038+9.2271E-4i

1.4250 3.3969+9.2481E-4i

1.4250 3.3995+9.5122E-4i

1.4250 3.4050+9.6448E-4i

1.4250 3.4054+9.7776E-4i

1.4250 3.4018+9.8986E-4i

1.4250 3.4037+0.0010026i

1.4250 3.4049+0.0010095i

1.5000 3.3839-2.2035E-4i

1.5000 3.3843-2.1631E-4i

1.5000 3.3826-2.1426E-4i

1.5000 3.3806-2.0229E-4i

1.5000 3.3839-1.9927E-4i

1.5000 3.3782-1.9218E-4i

1.5000 3.4159-1.8848E-4i

1.5000 3.4171-1.7627E-4i

1.5000 3.3826-1.7563E-4i

1.5000 3.4169-1.7451E-4i

1.5000 3.4172-1.6533E-4i

1.5000 3.4144-1.6478E-4i

1.5000 3.4165-1.6334E-4i

1.5000 3.4123-1.6099E-4i

1.5000 3.4152-1.5912E-4i

1.5000 3.3752-1.5852E-4i

1.5000 3.4165-1.5593E-4i

1.5000 3.4134-1.5575E-4i

1.5000 3.4171-1.5112E-4i

1.5000 3.3806-1.5056E-4i

1.5000 3.4169-1.4793E-4i

1.5000 3.4152-1.4606E-4i

1.5000 3.4087-1.4157E-4i

1.5000 3.4099-1.4112E-4i

1.5000 3.4111-1.3706E-4i

1.5000 3.4075-1.3318E-4i

1.5000 3.4144-1.3229E-4i

1.5000 3.4134-1.3156E-4i

1.5000 3.3720-1.2748E-4i

1.5000 3.4111-1.2732E-4i

1.5000 3.4066-1.2606E-4i

1.5000 3.3647-1.2526E-4i

1.5000 3.4159-1.2478E-4i

1.5000 3.3685-1.2471E-4i

1.5000 3.3781-1.2348E-4i

1.5000 3.4061-1.2265E-4i

1.5000 3.3752-1.2220E-4i

1.5000 3.3720-1.2093E-4i

1.5000 3.4123-1.1488E-4i

1.5000 3.4099-1.1307E-4i

1.5000 3.4066-1.0696E-4i

1.5000 3.4075-1.0635E-4i

1.5000 3.4087-1.0398E-4i

1.5000 3.4060-9.9962E-5i

1.5000 3.3685-9.4088E-5i

1.5000 3.4061-9.2533E-5i

1.5000 3.3607-8.7307E-5i

1.5000 3.3565-8.0993E-5i

1.5000 3.3607-7.7933E-5i

1.5000 3.3521-7.3555E-5i

1.5000 3.3647-6.5668E-5i

1.5000 3.3476-6.3931E-5i

1.5000 3.3565-6.0796E-5i

1.5000 3.3521-4.7199E-5i

1.5000 3.3475-3.9924E-5i

1.5000 3.3483-1.0860E-16i

1.5000 3.3483-6.5200E-17i

1.5000 3.3485+2.2785E-17i

1.5000 3.3485-2.2825E-16i

1.5000 3.3674+1.1957E-16i

1.5000 3.3674+7.4389E-17i

1.5000 3.3678+4.9864E-17i

1.5000 3.3678+1.7482E-16i

1.5000 3.3841-5.6205E-17i

1.5000 3.3841-5.1757E-17i

1.5000 3.3847-2.0448E-17i

1.5000 3.3847-4.6740E-17i

1.5000 3.3983-6.4336E-18i

1.5000 3.3983+1.9491E-18i

1.5000 3.3992+9.1882E-18i

1.5000 3.3992+3.3159E-17i

1.5000 3.4098+3.8938E-17i

1.5000 3.4098-5.8301E-18i

1.5000 3.4111+2.1419E-17i

1.5000 3.4111+2.2924E-17i

1.5000 3.4187-4.6275E-17i

1.5000 3.4187-7.6341E-17i

1.5000 3.4204+8.6602E-18i

1.5000 3.4204-8.0358E-19i

1.5000 3.4246-4.9333E-17i

1.5000 3.4246-1.3706E-16i

1.5000 3.4268-1.1570E-16i

1.5000 3.4272+9.8479E-17i

1.5000 3.4272+4.8317E-17i

1.5000 3.4315+1.8739E-17i

1.5000 3.4315-1.2126E-17i

1.5000 3.4335-8.3110E-18i

1.5000 3.4336-2.7526E-17i

1.5000 3.4340-4.9987E-18i

1.5000 3.4130+6.7757E-4i

1.5000 3.4140+6.9629E-4i

1.5000 3.4125+7.1299E-4i

1.5000 3.4154+7.2239E-4i

1.5000 3.4169+7.3910E-4i

1.5000 3.4129+7.5719E-4i

1.5000 3.4211+7.6153E-4i

1.5000 3.4249+7.6277E-4i

1.5000 3.3451+7.6362E-4i

1.5000 3.4184+7.6361E-4i

1.5000 3.4140+7.7247E-4i

1.5000 3.4233+7.8748E-4i

1.5000 3.4222+7.9099E-4i

1.5000 3.4153+7.9209E-4i

1.5000 3.4197+8.0533E-4i

1.5000 3.4261+8.1232E-4i

1.5000 3.4260+8.1553E-4i

1.5000 3.4168+8.1637E-4i

1.5000 3.4260+8.1835E-4i

1.5000 3.4197+8.2239E-4i

1.5000 3.4253+8.2338E-4i

1.5000 3.4183+8.2920E-4i

1.5000 3.4241+8.3017E-4i

1.5000 3.4253+8.4773E-4i

1.5000 3.4257+8.5511E-4i

1.5000 3.4257+8.6191E-4i

1.5000 3.4240+8.6231E-4i

1.5000 3.4221+8.6327E-4i

1.5000 3.4209+8.7644E-4i

1.5000 3.4231+8.8116E-4i

1.5000 3.3785+8.9060E-4i

1.5000 3.3570+9.0125E-4i

1.5000 3.3523+9.0249E-4i

1.5000 3.3661+9.0781E-4i

1.5000 3.3616+9.0876E-4i

1.5000 3.3704+9.1540E-4i

1.5000 3.4247+9.1778E-4i

1.5000 3.3822+9.3163E-4i

1.5000 3.3745+9.3535E-4i

1.5000 3.3615+9.3693E-4i

1.5000 3.3569+9.4326E-4i

1.5000 3.3660+9.4660E-4i

1.5000 3.3889+9.4682E-4i

1.5000 3.3744+9.5073E-4i

1.5000 3.3703+9.5230E-4i

1.5000 3.3522+9.6121E-4i

1.5000 3.3856+9.6932E-4i

1.5000 3.3919+9.7141E-4i

1.5000 3.3945+9.9170E-4i

1.5000 3.3488+9.9320E-4i

1.5000 3.3856+9.9606E-4i

1.5000 3.3821+9.9719E-4i

1.5000 3.3783+0.0010155i

1.5000 3.3965+0.0010544i

1.5000 3.3888+0.0010627i

1.5000 3.3917+0.0010910i

1.5000 3.3979+0.0011007i

1.5000 3.3983+0.0011154i

1.5000 3.3943+0.0011344i

1.5000 3.3964+0.0011472i

1.5000 3.3978+0.0011535i

1.5750 3.3744-2.5598E-4i

1.5750 3.3749-2.5082E-4i

1.5750 3.3730-2.5030E-4i

1.5750 3.3708-2.3790E-4i

1.5750 3.3744-2.3167E-4i

1.5750 3.3681-2.2774E-4i

1.5750 3.4099-2.1902E-4i

1.5750 3.3730-2.0550E-4i

1.5750 3.4112-2.0443E-4i

1.5750 3.4109-2.0246E-4i

1.5750 3.4082-1.9217E-4i

1.5750 3.4112-1.9174E-4i

1.5750 3.3649-1.8957E-4i

1.5750 3.4105-1.8958E-4i

1.5750 3.4059-1.8836E-4i

1.5750 3.4091-1.8521E-4i

1.5750 3.4071-1.8187E-4i

1.5750 3.4105-1.8103E-4i

1.5750 3.3708-1.7828E-4i

1.5750 3.4112-1.7521E-4i

1.5750 3.4109-1.7149E-4i

1.5750 3.4091-1.7017E-4i

1.5750 3.4018-1.6643E-4i

1.5750 3.4032-1.6595E-4i

1.5750 3.4046-1.6104E-4i

1.5750 3.4006-1.5675E-4i

1.5750 3.3613-1.5549E-4i

1.5750 3.3533-1.5455E-4i

1.5750 3.4081-1.5452E-4i

1.5750 3.4071-1.5419E-4i

1.5750 3.3574-1.5322E-4i

1.5750 3.4046-1.4982E-4i

1.5750 3.3649-1.4864E-4i

1.5750 3.3996-1.4830E-4i

1.5750 3.3681-1.4816E-4i

1.5750 3.3613-1.4798E-4i

1.5750 3.4098-1.4510E-4i

1.5750 3.3990-1.4283E-4i

1.5750 3.4059-1.3514E-4i

1.5750 3.4032-1.3361E-4i

1.5750 3.3996-1.2621E-4i

1.5750 3.4006-1.2606E-4i

1.5750 3.4018-1.2340E-4i

1.5750 3.3574-1.1804E-4i

1.5750 3.3988-1.1502E-4i

1.5750 3.3488-1.1173E-4i

1.5750 3.3990-1.0797E-4i

1.5750 3.3442-1.0497E-4i

1.5750 3.3488-1.0099E-4i

1.5750 3.3393-9.7052E-5i

1.5750 3.3343-8.6778E-5i

1.5750 3.3532-8.6282E-5i

1.5750 3.3442-8.1998E-5i

1.5750 3.3393-6.6799E-5i

1.5750 3.3343-5.9091E-5i

1.5750 3.3353+9.2347E-17i

1.5750 3.3353+1.9099E-16i

1.5750 3.3354+1.0551E-16i

1.5750 3.3354-8.6312E-17i

1.5750 3.3563+4.4322E-17i

1.5750 3.3563+5.6846E-17i

1.5750 3.3568+2.4394E-16i

1.5750 3.3568+2.9319E-18i

1.5750 3.3748-2.3086E-17i

1.5750 3.3748-1.1344E-17i

1.5750 3.3755+6.4134E-17i

1.5750 3.3755+5.4006E-17i

1.5750 3.3904+8.7581E-17i

1.5750 3.3904+1.7285E-16i

1.5750 3.3915-5.5267E-17i

1.5750 3.3915+1.9122E-17i

1.5750 3.4032-5.7159E-17i

1.5750 3.4032-1.2945E-17i

1.5750 3.4047+2.5915E-17i

1.5750 3.4047-4.9568E-17i

1.5750 3.4129-5.2063E-17i

1.5750 3.4129-5.6878E-18i

1.5750 3.4150+6.9363E-17i

1.5750 3.4150+3.0430E-17i

1.5750 3.4194-1.0398E-16i

1.5750 3.4194+8.3261E-17i

1.5750 3.4218-9.7395E-17i

1.5750 3.4225-6.6760E-17i

1.5750 3.4225-1.7639E-16i

1.5750 3.4273-4.5013E-17i

1.5750 3.4273-4.3321E-17i

1.5750 3.4296-1.0172E-17i

1.5750 3.4296+2.9241E-17i

1.5750 3.4301+5.6878E-18i

1.5750 3.4070+7.7082E-4i

1.5750 3.4081+7.9380E-4i

1.5750 3.4064+8.1135E-4i

1.5750 3.4096+8.2356E-4i

1.5750 3.4113+8.4277E-4i

1.5750 3.4068+8.6285E-4i

1.5750 3.4201+8.6506E-4i

1.5750 3.4159+8.6566E-4i

1.5750 3.4129+8.6962E-4i

1.5750 3.4080+8.8066E-4i

1.5750 3.3319+8.9385E-4i

1.5750 3.4183+8.9483E-4i

1.5750 3.4172+8.9952E-4i

1.5750 3.4095+9.0381E-4i

1.5750 3.4144+9.1764E-4i

1.5750 3.4215+9.2077E-4i

1.5750 3.4214+9.2496E-4i

1.5750 3.4213+9.2687E-4i

1.5750 3.4112+9.3100E-4i

1.5750 3.4207+9.3434E-4i

1.5750 3.4144+9.3692E-4i

1.5750 3.4192+9.4357E-4i

1.5750 3.4128+9.4609E-4i

1.5750 3.4207+9.6255E-4i

1.5750 3.4211+9.7136E-4i

1.5750 3.4211+9.7874E-4i

1.5750 3.4192+9.8033E-4i

1.5750 3.4171+9.8228E-4i

1.5750 3.4158+9.9890E-4i

1.5750 3.4182+0.0010024i

1.5750 3.3689+0.0010174i

1.5750 3.3451+0.0010355i

1.5750 3.3400+0.0010363i

1.5750 3.3552+0.0010406i

1.5750 3.3502+0.0010433i

1.5750 3.4199+0.0010436i

1.5750 3.3599+0.0010484i

1.5750 3.3730+0.0010629i

1.5750 3.3645+0.0010700i

1.5750 3.3501+0.0010757i

1.5750 3.3805+0.0010773i

1.5750 3.3450+0.0010837i

1.5750 3.3551+0.0010865i

1.5750 3.3644+0.0010888i

1.5750 3.3599+0.0010918i

1.5750 3.3838+0.0011044i

1.5750 3.3399+0.0011053i

1.5750 3.3769+0.0011059i

1.5750 3.3867+0.0011253i

1.5750 3.3768+0.0011355i

1.5750 3.3361+0.0011364i

1.5750 3.3729+0.0011397i

1.5750 3.3687+0.0011617i

1.5750 3.3890+0.0011958i

1.5750 3.3804+0.0012114i

1.5750 3.3836+0.0012414i

1.5750 3.3905+0.0012469i

1.5750 3.3910+0.0012628i

1.5750 3.3865+0.0012901i

1.5750 3.3889+0.0013027i

1.5750 3.3904+0.0013082i

1.6500 3.3645-2.9526E-4i

1.6500 3.3629-2.9011E-4i

1.6500 3.3650-2.8885E-4i

1.6500 3.3606-2.7729E-4i

1.6500 3.3576-2.6731E-4i

1.6500 3.3645-2.6739E-4i

1.6500 3.4035-2.5264E-4i

1.6500 3.3629-2.3848E-4i

1.6500 3.4049-2.3549E-4i

1.6500 3.4046-2.3327E-4i

1.6500 3.3540-2.2394E-4i

1.6500 3.4016-2.2241E-4i

1.6500 3.4050-2.2085E-4i

1.6500 3.3991-2.1860E-4i

1.6500 3.4042-2.1851E-4i

1.6500 3.4026-2.1397E-4i

1.6500 3.4004-2.1069E-4i

1.6500 3.3605-2.0904E-4i

1.6500 3.4042-2.0872E-4i

1.6500 3.4049-2.0174E-4i

1.6500 3.4046-1.9742E-4i

1.6500 3.4026-1.9678E-4i

1.6500 3.3947-1.9393E-4i

1.6500 3.3962-1.9343E-4i

1.6500 3.3412-1.8752E-4i

1.6500 3.3977-1.8757E-4i

1.6500 3.3501-1.8687E-4i

1.6500 3.3458-1.8527E-4i

1.6500 3.3933-1.8288E-4i

1.6500 3.4004-1.7918E-4i

1.6500 3.4016-1.7906E-4i

1.6500 3.3501-1.7829E-4i

1.6500 3.3540-1.7824E-4i

1.6500 3.3575-1.7565E-4i

1.6500 3.3977-1.7473E-4i

1.6500 3.3922-1.7307E-4i

1.6500 3.4035-1.6745E-4i

1.6500 3.3916-1.6512E-4i

1.6500 3.3991-1.5757E-4i

1.6500 3.3962-1.5638E-4i

1.6500 3.3933-1.4801E-4i

1.6500 3.3922-1.4768E-4i

1.6500 3.3458-1.4508E-4i

1.6500 3.3947-1.4499E-4i

1.6500 3.3364-1.3948E-4i

1.6500 3.3313-1.3232E-4i

1.6500 3.3914-1.3125E-4i

1.6500 3.3364-1.2724E-4i

1.6500 3.3915-1.2503E-4i

1.6500 3.3259-1.2400E-4i

1.6500 3.3204-1.1303E-4i

1.6500 3.3412-1.0978E-4i

1.6500 3.3312-1.0635E-4i

1.6500 3.3259-8.9482E-5i

1.6500 3.3204-8.1312E-5i

1.6500 3.3216+1.5245E-16i

1.6500 3.3216+2.1778E-17i

1.6500 3.3217-1.8331E-16i

1.6500 3.3217-1.5763E-16i

1.6500 3.3447+1.2099E-16i

1.6500 3.3447+1.5026E-16i

1.6500 3.3453+1.1738E-17i

1.6500 3.3453-1.0167E-17i

1.6500 3.3650-1.4140E-17i

1.6500 3.3650-1.8011E-17i

1.6500 3.3659-1.6272E-16i

1.6500 3.3659-1.8599E-16i

1.6500 3.3822-1.7372E-16i

1.6500 3.3822-1.1238E-16i

1.6500 3.3835-4.3332E-17i

1.6500 3.3835-6.2032E-17i

1.6500 3.3962-6.3339E-17i

1.6500 3.3962-9.5642E-17i

1.6500 3.3980+7.3139E-17i

1.6500 3.3980+4.6826E-17i

1.6500 3.4069+4.8275E-17i

1.6500 3.4069+4.2598E-17i

1.6500 3.4094+2.2813E-17i

1.6500 3.4094+2.8892E-17i

1.6500 3.4140+8.9045E-17i

1.6500 3.4140+3.9004E-17i

1.6500 3.4166+8.8429E-17i

1.6500 3.4176+2.9862E-17i

1.6500 3.4177-5.2575E-17i

1.6500 3.4229+2.0532E-17i

1.6500 3.4229+4.7406E-17i

1.6500 3.4255+4.2961E-17i

1.6500 3.4255+6.1643E-17i

1.6500 3.4261-2.5720E-17i

1.6500 3.4006+8.7105E-4i

1.6500 3.4019+8.9882E-4i

1.6500 3.4000+9.1705E-4i

1.6500 3.4036+9.3236E-4i

1.6500 3.4054+9.5435E-4i

1.6500 3.4153+9.7447E-4i

1.6500 3.4005+9.7663E-4i

1.6500 3.4106+9.7729E-4i

1.6500 3.4073+9.8350E-4i

1.6500 3.4018+9.9712E-4i

1.6500 3.4133+0.0010099i

1.6500 3.4120+0.0010159i

1.6500 3.4035+0.0010241i

1.6500 3.4167+0.0010368i

1.6500 3.3181+0.0010371i

1.6500 3.4090+0.0010383i

1.6500 3.4166+0.0010420i

1.6500 3.4166+0.0010428i

1.6500 3.4158+0.0010531i

1.6500 3.4054+0.0010543i

1.6500 3.4089+0.0010599i

1.6500 3.4143+0.0010651i

1.6500 3.4072+0.0010718i

1.6500 3.4158+0.0010855i

1.6500 3.4163+0.0010958i

1.6500 3.4163+0.0011040i

1.6500 3.4142+0.0011069i

1.6500 3.4119+0.0011100i

1.6500 3.4104+0.0011305i

1.6500 3.4131+0.0011325i

1.6500 3.3589+0.0011539i

1.6500 3.4150+0.0011786i

1.6500 3.3327+0.0011809i

1.6500 3.3270+0.0011812i

1.6500 3.3438+0.0011841i

1.6500 3.3383+0.0011890i

1.6500 3.3490+0.0011919i

1.6500 3.3635+0.0012040i

1.6500 3.3541+0.0012149i

1.6500 3.3717+0.0012172i

1.6500 3.3382+0.0012259i

1.6500 3.3326+0.0012359i

1.6500 3.3437+0.0012378i

1.6500 3.3540+0.0012376i

1.6500 3.3490+0.0012425i

1.6500 3.3754+0.0012468i

1.6500 3.3677+0.0012527i

1.6500 3.3269+0.0012617i

1.6500 3.3786+0.0012682i

1.6500 3.3677+0.0012852i

1.6500 3.3228+0.0012913i

1.6500 3.3633+0.0012930i

1.6500 3.3587+0.0013193i

1.6500 3.3811+0.0013471i

1.6500 3.3716+0.0013710i

1.6500 3.3752+0.0014025i

1.6500 3.3829+0.0014029i

1.6500 3.3834+0.0014200i

1.6500 3.3784+0.0014567i

1.6500 3.3811+0.0014691i

1.6500 3.3828+0.0014736i

1.7250 3.3541-3.3832E-4i

1.7250 3.3524-3.3388E-4i

1.7250 3.3546-3.3060E-4i

1.7250 3.3498-3.2067E-4i

1.7250 3.3465-3.1108E-4i

1.7250 3.3540-3.0654E-4i

1.7250 3.3969-2.8951E-4i

1.7250 3.3524-2.7475E-4i

1.7250 3.3984-2.6959E-4i

1.7250 3.3981-2.6709E-4i

1.7250 3.3426-2.6197E-4i

1.7250 3.3948-2.5564E-4i

1.7250 3.3985-2.5279E-4i

1.7250 3.3921-2.5185E-4i

1.7250 3.3976-2.5027E-4i

1.7250 3.3959-2.4550E-4i

1.7250 3.3498-2.4298E-4i

1.7250 3.3935-2.4233E-4i

1.7250 3.3976-2.3914E-4i

1.7250 3.3984-2.3083E-4i

1.7250 3.3959-2.2597E-4i

1.7250 3.3981-2.2585E-4i

1.7250 3.3287-2.2434E-4i

1.7250 3.3872-2.2420E-4i

1.7250 3.3889-2.2369E-4i

1.7250 3.3383-2.2175E-4i

1.7250 3.3337-2.2102E-4i

1.7250 3.3905-2.1677E-4i

1.7250 3.3383-2.1200E-4i

1.7250 3.3857-2.1170E-4i

1.7250 3.3426-2.1113E-4i

1.7250 3.3935-2.0661E-4i

1.7250 3.3465-2.0609E-4i

1.7250 3.3948-2.0601E-4i

1.7250 3.3905-2.0215E-4i

1.7250 3.3845-2.0053E-4i

1.7250 3.3968-1.9198E-4i

1.7250 3.3838-1.8964E-4i

1.7250 3.3921-1.8229E-4i

1.7250 3.3888-1.8149E-4i

1.7250 3.3336-1.7536E-4i

1.7250 3.3857-1.7232E-4i

1.7250 3.3845-1.7152E-4i

1.7250 3.3233-1.7071E-4i

1.7250 3.3872-1.6886E-4i

1.7250 3.3177-1.6317E-4i

1.7250 3.3233-1.5684E-4i

1.7250 3.3119-1.5454E-4i

1.7250 3.3836-1.4859E-4i

1.7250 3.3838-1.4380E-4i

1.7250 3.3058-1.4284E-4i

1.7250 3.3286-1.3632E-4i

1.7250 3.3177-1.3398E-4i

1.7250 3.3118-1.1536E-4i

1.7250 3.3058-1.0673E-4i

1.7250 3.3072-1.2380E-16i

1.7250 3.3072-1.6509E-16i

1.7250 3.3074-5.4190E-16i

1.7250 3.3074-2.3836E-16i

1.7250 3.3326+5.1604E-16i

1.7250 3.3326+1.6224E-16i

1.7250 3.3332+3.1387E-16i

1.7250 3.3332+6.2075E-17i

1.7250 3.3548+1.1013E-16i

1.7250 3.3548+1.2610E-16i

1.7250 3.3558-1.5919E-16i

1.7250 3.3559-1.5919E-16i

1.7250 3.3736+6.6554E-17i

1.7250 3.3736-9.0914E-17i

1.7250 3.3751-1.3741E-16i

1.7250 3.3751-6.0818E-17i

1.7250 3.3889-6.6630E-17i

1.7250 3.3889+8.6670E-17i

1.7250 3.3910-5.0606E-17i

1.7250 3.3910-1.7737E-16i

1.7250 3.4006-8.4024E-17i

1.7250 3.4006-7.0487E-17i

1.7250 3.4035+3.8801E-17i

1.7250 3.4035+4.5593E-17i

1.7250 3.4083+1.0164E-16i

1.7250 3.4083-9.2642E-17i

1.7250 3.4111-2.2192E-18i

1.7250 3.4126+7.7741E-18i

1.7250 3.4126+1.1893E-16i

1.7250 3.4184+2.0990E-17i

1.7250 3.4184-1.3225E-17i

1.7250 3.4212-7.1043E-17i

1.7250 3.4212+3.8999E-17i

1.7250 3.4219-6.5032E-17i

1.7250 3.3941+9.7833E-4i

1.7250 3.3955+0.0010114i

1.7250 3.3934+0.0010301i

1.7250 3.3974+0.0010489i

1.7250 3.3994+0.0010739i

1.7250 3.4102+0.0010910i

1.7250 3.4051+0.0010964i

1.7250 3.3939+0.0010986i

1.7250 3.4014+0.0011053i

1.7250 3.3954+0.0011219i

1.7250 3.4080+0.0011327i

1.7250 3.4066+0.0011402i

1.7250 3.3973+0.0011531i

1.7250 3.4118+0.0011603i

1.7250 3.4116+0.0011662i

1.7250 3.4117+0.0011668i

1.7250 3.4033+0.0011675i

1.7250 3.4108+0.0011797i

1.7250 3.3993+0.0011863i

1.7250 3.4032+0.0011914i

1.7250 3.3036+0.0011938i

1.7250 3.4091+0.0011949i

1.7250 3.4013+0.0012065i

1.7250 3.4108+0.0012166i

1.7250 3.4114+0.0012286i

1.7250 3.4113+0.0012376i

1.7250 3.4091+0.0012420i

1.7250 3.4065+0.0012463i

1.7250 3.4049+0.0012713i

1.7250 3.4078+0.0012715i

1.7250 3.3485+0.0013004i

1.7250 3.4099+0.0013227i

1.7250 3.3135+0.0013375i

1.7250 3.3197+0.0013378i

1.7250 3.3319+0.0013385i

1.7250 3.3259+0.0013459i

1.7250 3.3377+0.0013460i

1.7250 3.3535+0.0013548i

1.7250 3.3626+0.0013665i

1.7250 3.3432+0.0013705i

1.7250 3.3258+0.0013877i

1.7250 3.3431+0.0013975i

1.7250 3.3667+0.0013987i

1.7250 3.3196+0.0014001i

1.7250 3.3318+0.0014007i

1.7250 3.3376+0.0014045i

1.7250 3.3582+0.0014098i

1.7250 3.3702+0.0014203i

1.7250 3.3134+0.0014305i

1.7250 3.3581+0.0014450i

1.7250 3.3534+0.0014574i

1.7250 3.3089+0.0014579i

1.7250 3.3483+0.0014882i

1.7250 3.3730+0.0015083i

1.7250 3.3625+0.0015415i

1.7250 3.3749+0.0015688i

1.7250 3.3665+0.0015743i

1.7250 3.3756+0.0015866i

1.7250 3.3700+0.0016346i

1.7250 3.3729+0.0016465i

1.7250 3.3748+0.0016497i

1.8000 3.3432-3.8529E-4i

1.8000 3.3414-3.8177E-4i

1.8000 3.3438-3.7622E-4i

1.8000 3.3385-3.6819E-4i

1.8000 3.3350-3.5922E-4i

1.8000 3.3432-3.4923E-4i

1.8000 3.3900-3.2981E-4i

1.8000 3.3413-3.1444E-4i

1.8000 3.3917-3.0689E-4i

1.8000 3.3308-3.0398E-4i

1.8000 3.3913-3.0404E-4i

1.8000 3.3877-2.9194E-4i

1.8000 3.3847-2.8825E-4i

1.8000 3.3918-2.8770E-4i

1.8000 3.3907-2.8498E-4i

1.8000 3.3385-2.8026E-4i

1.8000 3.3889-2.7989E-4i

1.8000 3.3863-2.7693E-4i

1.8000 3.3907-2.7239E-4i

1.8000 3.3155-2.6519E-4i

1.8000 3.3916-2.6263E-4i

1.8000 3.3210-2.6062E-4i

1.8000 3.3261-2.6025E-4i

1.8000 3.3889-2.5784E-4i

1.8000 3.3794-2.5735E-4i

1.8000 3.3812-2.5686E-4i

1.8000 3.3913-2.5689E-4i

1.8000 3.3261-2.4920E-4i

1.8000 3.3830-2.4877E-4i

1.8000 3.3307-2.4746E-4i

1.8000 3.3778-2.4333E-4i

1.8000 3.3349-2.3959E-4i

1.8000 3.3863-2.3661E-4i

1.8000 3.3877-2.3546E-4i

1.8000 3.3830-2.3222E-4i

1.8000 3.3764-2.3086E-4i

1.8000 3.3899-2.1882E-4i

1.8000 3.3757-2.1649E-4i

1.8000 3.3847-2.0939E-4i

1.8000 3.3209-2.0903E-4i

1.8000 3.3812-2.0904E-4i

1.8000 3.3097-2.0558E-4i

1.8000 3.3778-1.9911E-4i

1.8000 3.3764-1.9788E-4i

1.8000 3.3035-1.9768E-4i

1.8000 3.3794-1.9512E-4i

1.8000 3.3097-1.8995E-4i

1.8000 3.2972-1.8880E-4i

1.8000 3.2905-1.7634E-4i

1.8000 3.3754-1.6689E-4i

1.8000 3.3154-1.6603E-4i

1.8000 3.3035-1.6505E-4i

1.8000 3.3756-1.6438E-4i

1.8000 3.2971-1.4455E-4i

1.8000 3.2905-1.3546E-4i

1.8000 3.2922+1.9874E-16i

1.8000 3.2922+1.6304E-16i

1.8000 3.2924+1.1876E-16i

1.8000 3.2924+1.6510E-16i

1.8000 3.3199-1.3687E-16i

1.8000 3.3199+3.6484E-16i

1.8000 3.3206+4.5498E-16i

1.8000 3.3206+1.4890E-16i

1.8000 3.3441-1.0919E-16i

1.8000 3.3441-1.4057E-16i

1.8000 3.3454-3.0616E-16i

1.8000 3.3454-3.2121E-16i

1.8000 3.3646-1.3240E-18i

1.8000 3.3646+2.3985E-17i

1.8000 3.3664-1.4128E-16i

1.8000 3.3665+7.0218E-17i

1.8000 3.3813-8.1205E-17i

1.8000 3.3813-4.4234E-17i

1.8000 3.3838-1.0293E-16i

1.8000 3.3838+1.0026E-16i

1.8000 3.3941+3.1719E-17i

1.8000 3.3941-2.1886E-17i

1.8000 3.3974+1.0196E-16i

1.8000 3.3974+1.0816E-16i

1.8000 3.4024+3.6469E-17i

1.8000 3.4024+1.0091E-16i

1.8000 3.4054-2.1485E-16i

1.8000 3.4074+4.7266E-17i

1.8000 3.4074+1.4493E-16i

1.8000 3.4137+5.0338E-18i

1.8000 3.4137+1.5367E-17i

1.8000 3.4168+1.0283E-16i

1.8000 3.4168+9.3678E-17i

1.8000 3.4176-4.8457E-17i

1.8000 3.3873+0.0010927i

1.8000 3.3888+0.0011317i

1.8000 3.3865+0.0011507i

1.8000 3.3909+0.0011732i

1.8000 3.3932+0.0012015i

1.8000 3.4050+0.0012146i

1.8000 3.3994+0.0012231i

1.8000 3.3871+0.0012289i

1.8000 3.3954+0.0012351i

1.8000 3.3888+0.0012550i

1.8000 3.4026+0.0012632i

1.8000 3.4011+0.0012724i

1.8000 3.3908+0.0012909i

1.8000 3.4068+0.0012913i

1.8000 3.4066+0.0012969i

1.8000 3.4067+0.0012991i

1.8000 3.3974+0.0013052i

1.8000 3.4057+0.0013140i

1.8000 3.3931+0.0013272i

1.8000 3.3974+0.0013315i

1.8000 3.4038+0.0013328i

1.8000 3.3952+0.0013501i

1.8000 3.4057+0.0013559i

1.8000 3.2885+0.0013642i

1.8000 3.4063+0.0013696i

1.8000 3.4062+0.0013796i

1.8000 3.4038+0.0013857i

1.8000 3.4010+0.0013914i

1.8000 3.4024+0.0014193i

1.8000 3.3992+0.0014214i

1.8000 3.3377+0.0014569i

1.8000 3.4047+0.0014759i

1.8000 3.3195+0.0015038i

1.8000 3.2994+0.0015053i

1.8000 3.3062+0.0015064i

1.8000 3.3259+0.0015109i

1.8000 3.3130+0.0015144i

1.8000 3.3432+0.0015155i

1.8000 3.3532+0.0015251i

1.8000 3.3319+0.0015369i

1.8000 3.3577+0.0015602i

1.8000 3.3129+0.0015614i

1.8000 3.3318+0.0015685i

1.8000 3.3194+0.0015755i

1.8000 3.3061+0.0015764i

1.8000 3.3483+0.0015773i

1.8000 3.3258+0.0015780i

1.8000 3.3615+0.0015815i

1.8000 3.2993+0.0016121i

1.8000 3.3483+0.0016151i

1.8000 3.3430+0.0016327i

1.8000 3.2944+0.0016366i

1.8000 3.3375+0.0016687i

1.8000 3.3646+0.0016794i

1.8000 3.3530+0.0017230i

1.8000 3.3668+0.0017444i

1.8000 3.3574+0.0017567i

1.8000 3.3675+0.0017625i

1.8000 3.3613+0.0018235i

1.8000 3.3645+0.0018347i

1.8000 3.3666+0.0018364i

1.8750 3.3318-4.3632E-4i

1.8750 3.3299-4.3398E-4i

1.8750 3.3325-4.2589E-4i

1.8750 3.3268-4.2004E-4i

1.8750 3.3229-4.1192E-4i

1.8750 3.3318-3.9566E-4i

1.8750 3.3828-3.7374E-4i

1.8750 3.3298-3.5771E-4i

1.8750 3.3183-3.5016E-4i

1.8750 3.3846-3.4754E-4i

1.8750 3.3842-3.4428E-4i

1.8750 3.3803-3.3144E-4i

1.8750 3.3771-3.2794E-4i

1.8750 3.3847-3.2569E-4i

1.8750 3.3268-3.2103E-4i

1.8750 3.3836-3.2277E-4i

1.8750 3.3816-3.1729E-4i

1.8750 3.3788-3.1463E-4i

1.8750 3.3017-3.1025E-4i

1.8750 3.3836-3.0862E-4i

1.8750 3.3077-3.0421E-4i

1.8750 3.3132-3.0255E-4i

1.8750 3.3846-2.9723E-4i

1.8750 3.3713-2.9350E-4i

1.8750 3.3733-2.9307E-4i

1.8750 3.3816-2.9253E-4i

1.8750 3.3842-2.9064E-4i

1.8750 3.3132-2.9003E-4i

1.8750 3.3183-2.8743E-4i

1.8750 3.3752-2.8370E-4i

1.8750 3.3695-2.7790E-4i

1.8750 3.3229-2.7630E-4i

1.8750 3.3788-2.6938E-4i

1.8750 3.3803-2.6751E-4i

1.8750 3.3752-2.6504E-4i

1.8750 3.3681-2.6422E-4i

1.8750 3.3827-2.4807E-4i

1.8750 3.3077-2.4620E-4i

1.8750 3.3672-2.4581E-4i

1.8750 3.2954-2.4425E-4i

1.8750 3.3732-2.3914E-4i

1.8750 3.3771-2.3899E-4i

1.8750 3.2887-2.3599E-4i

1.8750 3.3695-2.2848E-4i

1.8750 3.2818-2.2693E-4i

1.8750 3.2954-2.2671E-4i

1.8750 3.3680-2.2693E-4i

1.8750 3.3713-2.2388E-4i

1.8750 3.2746-2.1364E-4i

1.8750 3.2887-1.9969E-4i

1.8750 3.3017-1.9907E-4i

1.8750 3.3672-1.8684E-4i

1.8750 3.3669-1.8594E-4i

1.8750 3.2818-1.7716E-4i

1.8750 3.2746-1.6764E-4i

1.8750 3.2765+1.0820E-16i

1.8750 3.2765+1.5054E-17i

1.8750 3.2768-8.9709E-17i

1.8750 3.2768-2.0626E-16i

1.8750 3.3067-3.1160E-16i

1.8750 3.3067+2.3599E-17i

1.8750 3.3075+3.0624E-16i

1.8750 3.3075-5.4539E-16i

1.8750 3.3330-5.7170E-18i

1.8750 3.3330+2.3391E-17i

1.8750 3.3345-5.6616E-18i

1.8750 3.3345-4.5347E-17i

1.8750 3.3553-2.6346E-17i

1.8750 3.3553+1.2119E-16i

1.8750 3.3574-4.2150E-17i

1.8750 3.3574+1.2954E-16i

1.8750 3.3734+1.0889E-16i

1.8750 3.3734+1.0593E-16i

1.8750 3.3763+1.4656E-16i

1.8750 3.3763+1.5950E-16i

1.8750 3.3872+8.9500E-17i

1.8750 3.3872+5.5653E-17i

1.8750 3.3911-6.7181E-17i

1.8750 3.3911-5.4638E-17i

1.8750 3.3962+4.0796E-17i

1.8750 3.3962+1.3506E-17i

1.8750 3.3995+3.1665E-17i

1.8750 3.4019-1.1613E-16i

1.8750 3.4019-2.4563E-17i

1.8750 3.4089+3.9596E-17i

1.8750 3.4089+1.3460E-17i

1.8750 3.4123+6.1337E-17i

1.8750 3.4123-7.3328E-17i

1.8750 3.4132-9.8798E-17i

1.8750 3.3803+0.0012141i

1.8750 3.3820+0.0012597i

1.8750 3.3794+0.0012787i

1.8750 3.3843+0.0013054i

1.8750 3.3867+0.0013372i

1.8750 3.3997+0.0013452i

1.8750 3.3936+0.0013572i

1.8750 3.3800+0.0013676i

1.8750 3.3892+0.0013728i

1.8750 3.3819+0.0013966i

1.8750 3.3970+0.0014013i

1.8750 3.3954+0.0014125i

1.8750 3.4016+0.0014297i

1.8750 3.4014+0.0014350i

1.8750 3.3842+0.0014375i

1.8750 3.4015+0.0014390i

1.8750 3.3914+0.0014513i

1.8750 3.4004+0.0014561i

1.8750 3.3866+0.0014767i

1.8750 3.3984+0.0014788i

1.8750 3.3913+0.0014800i

1.8750 3.3890+0.0015028i

1.8750 3.4004+0.0015032i

1.8750 3.4010+0.0015189i

1.8750 3.4010+0.0015298i

1.8750 3.3983+0.0015378i

1.8750 3.3952+0.0015452i

1.8750 3.2728+0.0015490i

1.8750 3.3968+0.0015760i

1.8750 3.3933+0.0015806i

1.8750 3.3265+0.0016235i

1.8750 3.3993+0.0016382i

1.8750 3.3067+0.0016804i

1.8750 3.2847+0.0016850i

1.8750 3.3325+0.0016859i

1.8750 3.3136+0.0016866i

1.8750 3.2921+0.0016869i

1.8750 3.3434+0.0016931i

1.8750 3.2995+0.0016947i

1.8750 3.3202+0.0017144i

1.8750 3.3483+0.0017311i

1.8750 3.2994+0.0017471i

1.8750 3.3201+0.0017508i

1.8750 3.3526+0.0017518i

1.8750 3.3381+0.0017552i

1.8750 3.3066+0.0017623i

1.8750 3.3135+0.0017631i

1.8750 3.2921+0.0017652i

1.8750 3.3380+0.0017955i

1.8750 3.2846+0.0018067i

1.8750 3.3323+0.0018192i

1.8750 3.2793+0.0018276i

1.8750 3.3560+0.0018603i

1.8750 3.3263+0.0018608i

1.8750 3.3432+0.0019155i

1.8750 3.3583+0.0019297i

1.8750 3.3591+0.0019473i

1.8750 3.3480+0.0019498i

1.8750 3.3523+0.0020235i

1.8750 3.3559+0.0020338i

1.8750 3.3582+0.0020338i

1.9500 3.3200-4.9166E-4i

1.9500 3.3179-4.9068E-4i

1.9500 3.3208-4.7979E-4i

1.9500 3.3146-4.7638E-4i

1.9500 3.3104-4.6936E-4i

1.9500 3.3200-4.4606E-4i

1.9500 3.3753-4.2145E-4i

1.9500 3.3179-4.0470E-4i

1.9500 3.3054-4.0059E-4i

1.9500 3.3773-3.9169E-4i

1.9500 3.3769-3.8794E-4i

1.9500 3.3726-3.7426E-4i

1.9500 3.3691-3.7106E-4i

1.9500 3.3774-3.6690E-4i

1.9500 3.3145-3.6543E-4i

1.9500 3.3762-3.6378E-4i

1.9500 3.2874-3.5966E-4i

1.9500 3.3741-3.5785E-4i

1.9500 3.3710-3.5554E-4i

1.9500 3.2939-3.5190E-4i

1.9500 3.2999-3.4889E-4i

1.9500 3.3762-3.4794E-4i

1.9500 3.3773-3.3477E-4i

1.9500 3.2999-3.3470E-4i

1.9500 3.3629-3.3275E-4i

1.9500 3.3650-3.3242E-4i

1.9500 3.3054-3.3119E-4i

1.9500 3.3741-3.3019E-4i

1.9500 3.3769-3.2722E-4i

1.9500 3.3671-3.2166E-4i

1.9500 3.3103-3.1633E-4i

1.9500 3.3609-3.1551E-4i

1.9500 3.3710-3.0506E-4i

1.9500 3.3726-3.0228E-4i

1.9500 3.3593-3.0079E-4i

1.9500 3.3671-3.0074E-4i

1.9500 3.2938-2.8697E-4i

1.9500 3.2805-2.8687E-4i

1.9500 3.3752-2.7983E-4i

1.9500 3.2733-2.7826E-4i

1.9500 3.3584-2.7771E-4i

1.9500 3.3650-2.7189E-4i

1.9500 3.3691-2.7119E-4i

1.9500 3.2658-2.6904E-4i

1.9500 3.2805-2.6728E-4i

1.9500 3.3609-2.6055E-4i

1.9500 3.3593-2.5882E-4i

1.9500 3.3629-2.5523E-4i

1.9500 3.2580-2.5483E-4i

1.9500 3.2733-2.3803E-4i

1.9500 3.2874-2.3557E-4i

1.9500 3.2657-2.1330E-4i

1.9500 3.3584-2.1128E-4i

1.9500 3.3581-2.0542E-4i

1.9500 3.2579-2.0333E-4i

1.9500 3.2602-8.5194E-17i

1.9500 3.2602-1.0327E-17i

1.9500 3.2605-6.3060E-17i

1.9500 3.2605-7.5897E-17i

1.9500 3.2929-2.7727E-16i

1.9500 3.2929+9.4895E-17i

1.9500 3.2938-1.6808E-16i

1.9500 3.2939+3.5212E-16i

1.9500 3.3214+1.7061E-16i

1.9500 3.3214+1.3552E-16i

1.9500 3.3231+1.0197E-16i

1.9500 3.3231+7.6473E-17i

1.9500 3.3456+2.0171E-16i

1.9500 3.3456+1.8742E-16i

1.9500 3.3480-1.8095E-17i

1.9500 3.3480-1.2730E-16i

1.9500 3.3652+1.0802E-16i

1.9500 3.3652+5.3567E-17i

1.9500 3.3685-9.8203E-17i

1.9500 3.3685-5.0486E-17i

1.9500 3.3801-1.6095E-17i

1.9500 3.3801+9.4011E-18i

1.9500 3.3846+1.8269E-16i

1.9500 3.3846+6.1225E-17i

1.9500 3.3898+1.3011E-16i

1.9500 3.3898-1.2253E-16i

1.9500 3.3933+8.7709E-17i

1.9500 3.3964+1.0424E-16i

1.9500 3.3964-1.3822E-16i

1.9500 3.4039-1.0867E-16i

1.9500 3.4039-1.2696E-16i

1.9500 3.4077+1.0882E-17i

1.9500 3.4077+8.3596E-17i

1.9500 3.4087-7.9545E-17i

1.9500 3.3730+0.0013426i

1.9500 3.3749+0.0013955i

1.9500 3.3721+0.0014144i

1.9500 3.3774+0.0014455i

1.9500 3.3801+0.0014810i

1.9500 3.3942+0.0014828i

1.9500 3.3876+0.0014987i

1.9500 3.3728+0.0015146i

1.9500 3.3827+0.0015185i

1.9500 3.3748+0.0015467i

1.9500 3.3913+0.0015471i

1.9500 3.3895+0.0015603i

1.9500 3.3963+0.0015754i

1.9500 3.3960+0.0015803i

1.9500 3.3961+0.0015863i

1.9500 3.3773+0.0015931i

1.9500 3.3852+0.0016059i

1.9500 3.3950+0.0016058i

1.9500 3.3928+0.0016330i

1.9500 3.3800+0.0016351i

1.9500 3.3851+0.0016370i

1.9500 3.3950+0.0016585i

1.9500 3.3826+0.0016643i

1.9500 3.3957+0.0016763i

1.9500 3.3956+0.0016883i

1.9500 3.3927+0.0016984i

1.9500 3.3894+0.0017076i

1.9500 3.3911+0.0017414i

1.9500 3.2564+0.0017483i

1.9500 3.3872+0.0017491i

1.9500 3.3149+0.0018003i

1.9500 3.3938+0.0018096i

1.9500 3.3214+0.0018664i

1.9500 3.2934+0.0018683i

1.9500 3.3333+0.0018704i

1.9500 3.3009+0.0018735i

1.9500 3.2695+0.0018768i

1.9500 3.2775+0.0018797i

1.9500 3.2856+0.0018869i

1.9500 3.3080+0.0019029i

1.9500 3.3386+0.0019116i

1.9500 3.3433+0.0019312i

1.9500 3.3275+0.0019437i

1.9500 3.3079+0.0019446i

1.9500 3.2854+0.0019452i

1.9500 3.3007+0.0019600i

1.9500 3.2932+0.0019612i

1.9500 3.2775+0.0019666i

1.9500 3.3274+0.0019863i

1.9500 3.2693+0.0020145i

1.9500 3.3212+0.0020170i

1.9500 3.2636+0.0020311i

1.9500 3.3470+0.0020510i

1.9500 3.3147+0.0020647i

1.9500 3.3331+0.0021190i

1.9500 3.3496+0.0021246i

1.9500 3.3505+0.0021406i

1.9500 3.3383+0.0021534i

1.9500 3.3430+0.0022345i

1.9500 3.3495+0.0022416i

1.9500 3.3469+0.0022438i

2.0250 3.3054-5.5204E-4i

2.0250 3.3077-5.5152E-4i

2.0250 3.3085-5.3810E-4i

2.0250 3.3019-5.3740E-4i

2.0250 3.2973-5.3174E-4i

2.0250 3.3077-5.0061E-4i

2.0250 3.3675-4.7308E-4i

2.0250 3.2920-4.5540E-4i

2.0250 3.3054-4.5555E-4i

2.0250 3.3697-4.3950E-4i

2.0250 3.3692-4.3517E-4i

2.0250 3.3647-4.2058E-4i

2.0250 3.3609-4.1774E-4i

2.0250 3.2725-4.1354E-4i

2.0250 3.3018-4.1363E-4i

2.0250 3.3698-4.1144E-4i

2.0250 3.3685-4.0813E-4i

2.0250 3.2795-4.0386E-4i

2.0250 3.3662-4.0173E-4i

2.0250 3.2860-3.9949E-4i

2.0250 3.3629-3.9980E-4i

2.0250 3.3685-3.9048E-4i

2.0250 3.2860-3.8340E-4i

2.0250 3.2919-3.7893E-4i

2.0250 3.3697-3.7535E-4i

2.0250 3.3541-3.7520E-4i

2.0250 3.3564-3.7506E-4i

2.0250 3.3662-3.7100E-4i

2.0250 3.3692-3.6674E-4i

2.0250 3.3587-3.6280E-4i

2.0250 3.2973-3.5981E-4i

2.0250 3.3520-3.5625E-4i

2.0250 3.3629-3.4379E-4i

2.0250 3.3503-3.4074E-4i

2.0250 3.3647-3.3992E-4i

2.0250 3.3587-3.3943E-4i

2.0250 3.2650-3.3359E-4i

2.0250 3.2794-3.3143E-4i

2.0250 3.2572-3.2461E-4i

2.0250 3.2491-3.1526E-4i

2.0250 3.3675-3.1417E-4i

2.0250 3.3493-3.1233E-4i

2.0250 3.2650-3.1178E-4i

2.0250 3.3564-3.0740E-4i

2.0250 3.3609-3.0609E-4i

2.0250 3.2406-2.9994E-4i

2.0250 3.3520-2.9540E-4i

2.0250 3.3503-2.9373E-4i

2.0250 3.3541-2.8929E-4i

2.0250 3.2572-2.8021E-4i

2.0250 3.2724-2.7562E-4i

2.0250 3.2490-2.5302E-4i

2.0250 3.2406-2.4261E-4i

2.0250 3.3492-2.3781E-4i

2.0250 3.3489-2.2488E-4i

2.0250 3.2432+8.1310E-17i

2.0250 3.2432+1.8825E-16i

2.0250 3.2435+1.1886E-16i

2.0250 3.2435+1.8474E-16i

2.0250 3.2786-2.0531E-16i

2.0250 3.2786-9.0765E-17i

2.0250 3.2797-2.4884E-16i

2.0250 3.2797-2.8264E-16i

2.0250 3.3094-2.0338E-17i

2.0250 3.3094-3.0684E-17i

2.0250 3.3114+1.4332E-17i

2.0250 3.3114+5.7077E-17i

2.0250 3.3355-3.8696E-17i

2.0250 3.3355-2.0984E-16i

2.0250 3.3383-1.1178E-16i

2.0250 3.3383-1.1705E-16i

2.0250 3.3567-2.9699E-17i

2.0250 3.3567-1.1926E-16i

2.0250 3.3605+6.3147E-17i

2.0250 3.3605+4.2403E-17i

2.0250 3.3728-1.7814E-16i

2.0250 3.3728-2.2580E-16i

2.0250 3.3779-3.3773E-17i

2.0250 3.3779-8.5539E-17i

2.0250 3.3832-3.6575E-17i

2.0250 3.3832+6.5984E-17i

2.0250 3.3869+2.1012E-16i

2.0250 3.3906-1.9270E-17i

2.0250 3.3906-1.6956E-16i

2.0250 3.3988-2.8465E-17i

2.0250 3.3988-8.2013E-17i

2.0250 3.4030+6.0976E-17i

2.0250 3.4030+1.0609E-16i

2.0250 3.4041-1.5345E-16i

2.0250 3.3656+0.0014783i

2.0250 3.3676+0.0015392i

2.0250 3.3645+0.0015578i

2.0250 3.3704+0.0015935i

2.0250 3.3886+0.0016273i

2.0250 3.3733+0.0016329i

2.0250 3.3814+0.0016476i

2.0250 3.3653+0.0016701i

2.0250 3.3761+0.0016721i

2.0250 3.3855+0.0017004i

2.0250 3.3675+0.0017055i

2.0250 3.3835+0.0017159i

2.0250 3.3908+0.0017285i

2.0250 3.3905+0.0017327i

2.0250 3.3907+0.0017411i

2.0250 3.3702+0.0017575i

2.0250 3.3894+0.0017632i

2.0250 3.3788+0.0017689i

2.0250 3.3870+0.0017951i

2.0250 3.3731+0.0018023i

2.0250 3.3787+0.0018024i

2.0250 3.3894+0.0018218i

2.0250 3.3760+0.0018349i

2.0250 3.3902+0.0018419i

2.0250 3.3901+0.0018549i

2.0250 3.3870+0.0018673i

2.0250 3.3833+0.0018786i

2.0250 3.3852+0.0019155i

2.0250 3.3810+0.0019267i

2.0250 3.2394+0.0019628i

2.0250 3.3029+0.0019875i

2.0250 3.3881+0.0019899i

2.0250 3.3100+0.0020570i

2.0250 3.3229+0.0020572i

2.0250 3.2795+0.0020676i

2.0250 3.2877+0.0020718i

2.0250 3.2537+0.0020808i

2.0250 3.2624+0.0020849i

2.0250 3.2711+0.0020912i

2.0250 3.3287+0.0021016i

2.0250 3.2954+0.0021027i

2.0250 3.3337+0.0021194i

2.0250 3.3166+0.0021427i

2.0250 3.2953+0.0021499i

2.0250 3.2710+0.0021558i

2.0250 3.2875+0.0021690i

2.0250 3.2794+0.0021725i

2.0250 3.2623+0.0021810i

2.0250 3.3165+0.0021873i

2.0250 3.3097+0.0022261i

2.0250 3.2535+0.0022360i

2.0250 3.2473+0.0022474i

2.0250 3.3378+0.0022516i

2.0250 3.3027+0.0022803i

2.0250 3.3407+0.0023290i

2.0250 3.3226+0.0023335i

2.0250 3.3416+0.0023416i

2.0250 3.3283+0.0023674i

2.0250 3.3334+0.0024565i

2.0250 3.3405+0.0024599i

2.0250 3.3377+0.0024646i

2.1000 3.2925-6.1823E-4i

2.1000 3.2950-6.1608E-4i

2.1000 3.2886-6.0327E-4i

2.1000 3.2958-6.0097E-4i

2.1000 3.2837-5.9923E-4i

2.1000 3.2950-5.5945E-4i

2.1000 3.3595-5.2879E-4i

2.1000 3.2780-5.1472E-4i

2.1000 3.2925-5.1041E-4i

2.1000 3.3618-4.9111E-4i

2.1000 3.3614-4.8611E-4i

2.1000 3.2570-4.7199E-4i

2.1000 3.3564-4.7060E-4i

2.1000 3.3524-4.6814E-4i

2.1000 3.2886-4.6576E-4i

2.1000 3.2645-4.6031E-4i

2.1000 3.3620-4.5944E-4i

2.1000 3.3606-4.5595E-4i

2.1000 3.2715-4.5451E-4i

2.1000 3.3581-4.4906E-4i

2.1000 3.3545-4.4752E-4i

2.1000 3.3606-4.3636E-4i

2.1000 3.2715-4.3631E-4i

2.1000 3.2779-4.3078E-4i

2.1000 3.3475-4.2109E-4i

2.1000 3.3451-4.2095E-4i

2.1000 3.3618-4.1911E-4i

2.1000 3.3581-4.1507E-4i

2.1000 3.3613-4.0931E-4i

2.1000 3.3500-4.0721E-4i

2.1000 3.2837-4.0687E-4i

2.1000 3.3428-4.0023E-4i

2.1000 3.3545-3.8568E-4i

2.1000 3.2489-3.8453E-4i

2.1000 3.3409-3.8422E-4i

2.1000 3.3500-3.8123E-4i

2.1000 3.3564-3.8056E-4i

2.1000 3.2645-3.7974E-4i

2.1000 3.2405-3.7516E-4i

2.1000 3.2317-3.6567E-4i

2.1000 3.2489-3.6032E-4i

2.1000 3.3594-3.5120E-4i

2.1000 3.3398-3.4986E-4i

2.1000 3.2226-3.4899E-4i

2.1000 3.3475-3.4576E-4i

2.1000 3.3523-3.4381E-4i

2.1000 3.3427-3.3313E-4i

2.1000 3.3409-3.3180E-4i

2.1000 3.2405-3.2631E-4i

2.1000 3.3450-3.2614E-4i

2.1000 3.2569-3.1934E-4i

2.1000 3.2317-2.9639E-4i

2.1000 3.2225-2.8552E-4i

2.1000 3.3397-2.6653E-4i

2.1000 3.3394-2.4368E-4i

2.1000 3.2256-8.6923E-17i

2.1000 3.2256-1.0736E-16i

2.1000 3.2258-3.2432E-16i

2.1000 3.2258-1.3146E-16i

2.1000 3.2637+1.9314E-16i

2.1000 3.2637+8.4994E-18i

2.1000 3.2649+1.7293E-16i

2.1000 3.2649-5.7800E-17i

2.1000 3.2969+1.4206E-16i

2.1000 3.2970+1.7146E-16i

2.1000 3.2992-1.5118E-16i

2.1000 3.2992-1.0344E-16i

2.1000 3.3251-5.7716E-17i

2.1000 3.3251-1.1144E-17i

2.1000 3.3283-1.4139E-16i

2.1000 3.3283+6.0253E-17i

2.1000 3.3479-1.0020E-16i

2.1000 3.3479-7.8165E-17i

2.1000 3.3522-1.0437E-16i

2.1000 3.3522+1.8541E-17i

2.1000 3.3652+4.9621E-17i

2.1000 3.3652+8.0006E-17i

2.1000 3.3710-2.2518E-16i

2.1000 3.3710-1.8376E-16i

2.1000 3.3763-1.4236E-16i

2.1000 3.3763-4.4303E-17i

2.1000 3.3803-3.1699E-17i

2.1000 3.3847+2.0330E-16i

2.1000 3.3847-1.0217E-16i

2.1000 3.3936-1.3751E-16i

2.1000 3.3936-1.2509E-16i

2.1000 3.3981+2.0551E-16i

2.1000 3.3981+4.5213E-17i

2.1000 3.3993+1.0832E-16i

2.1000 3.3579+0.0016212i

2.1000 3.3601+0.0016908i

2.1000 3.3568+0.0017087i

2.1000 3.3631+0.0017494i

2.1000 3.3828+0.0017785i

2.1000 3.3663+0.0017928i

2.1000 3.3750+0.0018037i

2.1000 3.3694+0.0018336i

2.1000 3.3575+0.0018342i

2.1000 3.3795+0.0018611i

2.1000 3.3600+0.0018728i

2.1000 3.3773+0.0018792i

2.1000 3.3852+0.0018886i

2.1000 3.3850+0.0018922i

2.1000 3.3851+0.0019031i

2.1000 3.3837+0.0019279i

2.1000 3.3630+0.0019308i

2.1000 3.3722+0.0019404i

2.1000 3.3811+0.0019651i

2.1000 3.3721+0.0019762i

2.1000 3.3661+0.0019782i

2.1000 3.3837+0.0019928i

2.1000 3.3692+0.0020143i

2.1000 3.3846+0.0020153i

2.1000 3.3845+0.0020295i

2.1000 3.3811+0.0020445i

2.1000 3.3772+0.0020581i

2.1000 3.3792+0.0020983i

2.1000 3.3747+0.0021134i

2.1000 3.3823+0.0021790i

2.1000 3.2905+0.0021851i

2.1000 3.2218+0.0021928i

2.1000 3.3121+0.0022532i

2.1000 3.2981+0.0022577i

2.1000 3.2652+0.0022787i

2.1000 3.2740+0.0022816i

2.1000 3.2373+0.0022975i

2.1000 3.3184+0.0023010i

2.1000 3.2467+0.0023028i

2.1000 3.2561+0.0023078i

2.1000 3.2824+0.0023138i

2.1000 3.3239+0.0023164i

2.1000 3.3053+0.0023522i

2.1000 3.2823+0.0023670i

2.1000 3.2559+0.0023791i

2.1000 3.2738+0.0023903i

2.1000 3.2651+0.0023963i

2.1000 3.3052+0.0023987i

2.1000 3.2466+0.0024085i

2.1000 3.2979+0.0024467i

2.1000 3.3284+0.0024620i

2.1000 3.2371+0.0024713i

2.1000 3.2304+0.0024765i

2.1000 3.2902+0.0025078i

2.1000 3.3315+0.0025427i

2.1000 3.3325+0.0025495i

2.1000 3.3118+0.0025590i

2.1000 3.3180+0.0025918i

2.1000 3.3313+0.0026885i

2.1000 3.3235+0.0026894i

2.1000 3.3282+0.0026960i

2.1750 3.2791-6.8942E-4i

2.1750 3.2817-6.8551E-4i

2.1750 3.2749-6.7415E-4i

2.1750 3.2697-6.7202E-4i

2.1750 3.2827-6.6858E-4i

2.1750 3.2817-6.2271E-4i

2.1750 3.3512-5.8873E-4i

2.1750 3.2634-5.7869E-4i

2.1750 3.2790-5.6940E-4i

2.1750 3.3537-5.4670E-4i

2.1750 3.3532-5.4090E-4i

2.1750 3.2408-5.3512E-4i

2.1750 3.3479-5.2444E-4i

2.1750 3.3435-5.2237E-4i

2.1750 3.2749-5.2197E-4i

2.1750 3.2490-5.2146E-4i

2.1750 3.2565-5.1412E-4i

2.1750 3.3538-5.1105E-4i

2.1750 3.3524-5.0738E-4i

2.1750 3.3497-4.9996E-4i

2.1750 3.3458-4.9885E-4i

2.1750 3.2565-4.9355E-4i

2.1750 3.2634-4.8692E-4i

2.1750 3.3523-4.8570E-4i

2.1750 3.3384-4.7063E-4i

2.1750 3.3357-4.7008E-4i

2.1750 3.3537-4.6614E-4i

2.1750 3.3497-4.6253E-4i

2.1750 3.2696-4.5761E-4i

2.1750 3.3532-4.5504E-4i

2.1750 3.3410-4.5502E-4i

2.1750 3.3332-4.4749E-4i

2.1750 3.2322-4.3979E-4i

2.1750 3.2489-4.3212E-4i

2.1750 3.3312-4.3140E-4i

2.1750 3.3458-4.3085E-4i

2.1750 3.2231-4.3003E-4i

2.1750 3.3410-4.2625E-4i

2.1750 3.3479-4.2432E-4i

2.1750 3.2136-4.2035E-4i

2.1750 3.2322-4.1300E-4i

2.1750 3.2038-4.0201E-4i

2.1750 3.3511-3.9101E-4i

2.1750 3.3300-3.9051E-4i

2.1750 3.3383-3.8707E-4i

2.1750 3.3435-3.8444E-4i

2.1750 3.2231-3.7644E-4i

2.1750 3.3332-3.7379E-4i

2.1750 3.3312-3.7319E-4i

2.1750 3.2408-3.6683E-4i

2.1750 3.3357-3.6586E-4i

2.1750 3.2136-3.4343E-4i

2.1750 3.2038-3.3203E-4i

2.1750 3.3299-2.9763E-4i

2.1750 3.3296-2.6098E-4i

2.1750 3.2072-8.3813E-16i

2.1750 3.2072-2.5773E-16i

2.1750 3.2075+2.6073E-16i

2.1750 3.2075+2.1341E-16i

2.1750 3.2483-7.3840E-18i

2.1750 3.2483+3.8283E-16i

2.1750 3.2496-2.1871E-16i

2.1750 3.2496+3.5117E-17i

2.1750 3.2840+1.0326E-16i

2.1750 3.2840+1.4286E-16i

2.1750 3.2865-3.0706E-16i

2.1750 3.2865-3.8905E-16i

2.1750 3.3142-5.1075E-17i

2.1750 3.3142+9.5649E-17i

2.1750 3.3179-5.2045E-16i

2.1750 3.3179-2.7026E-17i

2.1750 3.3387+5.5874E-17i

2.1750 3.3387-1.7255E-17i

2.1750 3.3437+7.9139E-17i

2.1750 3.3437-6.8126E-17i

2.1750 3.3573+5.5683E-17i

2.1750 3.3573+3.4799E-18i

2.1750 3.3639+4.9672E-16i

2.1750 3.3639+4.1163E-16i

2.1750 3.3692-9.0124E-17i

2.1750 3.3692+1.6804E-17i

2.1750 3.3735-1.5506E-16i

2.1750 3.3787+6.6774E-17i

2.1750 3.3787-3.7484E-17i

2.1750 3.3882-6.8303E-17i

2.1750 3.3882-1.4651E-16i

2.1750 3.3931+1.0252E-16i

2.1750 3.3931-9.2883E-17i

2.1750 3.3945-1.9752E-16i

2.1750 3.3499+0.0017713i

2.1750 3.3524+0.0018504i

2.1750 3.3487+0.0018673i

2.1750 3.3557+0.0019132i

2.1750 3.3769+0.0019363i

2.1750 3.3591+0.0019608i

2.1750 3.3686+0.0019671i

2.1750 3.3624+0.0020029i

2.1750 3.3496+0.0020068i

2.1750 3.3733+0.0020291i

2.1750 3.3523+0.0020487i

2.1750 3.3710+0.0020500i

2.1750 3.3796+0.0020558i

2.1750 3.3792+0.0020586i

2.1750 3.3794+0.0020722i

2.1750 3.3779+0.0021000i

2.1750 3.3555+0.0021130i

2.1750 3.3655+0.0021201i

2.1750 3.3751+0.0021429i

2.1750 3.3654+0.0021582i

2.1750 3.3589+0.0021628i

2.1750 3.3779+0.0021716i

2.1750 3.3788+0.0021966i

2.1750 3.3622+0.0022025i

2.1750 3.3787+0.0022119i

2.1750 3.3751+0.0022298i

2.1750 3.3708+0.0022459i

2.1750 3.3730+0.0022895i

2.1750 3.3681+0.0023091i

2.1750 3.3764+0.0023768i

2.1750 3.2777+0.0023933i

2.1750 3.2035+0.0024389i

2.1750 3.3010+0.0024585i

2.1750 3.2859+0.0024686i

2.1750 3.2504+0.0025018i

2.1750 3.2599+0.0025030i

2.1750 3.3078+0.0025099i

2.1750 3.3138+0.0025221i

2.1750 3.2203+0.0025270i

2.1750 3.2304+0.0025336i

2.1750 3.2689+0.0025363i

2.1750 3.2406+0.0025370i

2.1750 3.2936+0.0025724i

2.1750 3.2689+0.0025959i

2.1750 3.2404+0.0026153i

2.1750 3.2936+0.0026204i

2.1750 3.2597+0.0026240i

2.1750 3.2502+0.0026330i

2.1750 3.2303+0.0026494i

2.1750 3.2857+0.0026789i

2.1750 3.3186+0.0026820i

2.1750 3.2129+0.0027189i

2.1750 3.2201+0.0027208i

2.1750 3.2774+0.0027474i

2.1750 3.3232+0.0027634i

2.1750 3.3220+0.0027657i

2.1750 3.3007+0.0027954i

2.1750 3.3074+0.0028264i

2.1750 3.3219+0.0029275i

2.1750 3.3134+0.0029330i

2.1750 3.3185+0.0029380i

2.2500 3.2652-7.6576E-4i

2.2500 3.2680-7.5995E-4i

2.2500 3.2551-7.5029E-4i

2.2500 3.2607-7.5021E-4i

2.2500 3.2690-7.4108E-4i

2.2500 3.2680-6.9053E-4i

2.2500 3.3426-6.5305E-4i

2.2500 3.2484-6.4745E-4i

2.2500 3.2651-6.3266E-4i

2.2500 3.3453-6.0642E-4i

2.2500 3.2241-6.0307E-4i

2.2500 3.3448-5.9967E-4i

2.2500 3.2328-5.8751E-4i

2.2500 3.2607-5.8241E-4i

2.2500 3.3391-5.8225E-4i

2.2500 3.3344-5.8058E-4i

2.2500 3.2409-5.7845E-4i

2.2500 3.3455-5.6636E-4i

2.2500 3.3439-5.6254E-4i

2.2500 3.2409-5.5524E-4i

2.2500 3.3410-5.5455E-4i

2.2500 3.3369-5.5388E-4i

2.2500 3.2484-5.4749E-4i

2.2500 3.3438-5.3862E-4i

2.2500 3.3288-5.2379E-4i

2.2500 3.3260-5.2266E-4i

2.2500 3.3453-5.1655E-4i

2.2500 3.3410-5.1349E-4i

2.2500 3.2550-5.1216E-4i

2.2500 3.3317-5.0635E-4i

2.2500 3.3447-5.0404E-4i

2.2500 3.2148-4.9944E-4i

2.2500 3.3233-4.9807E-4i

2.2500 3.2051-4.8928E-4i

2.2500 3.2328-4.8875E-4i

2.2500 3.3211-4.8238E-4i

2.2500 3.1949-4.7936E-4i

2.2500 3.3368-4.7941E-4i

2.2500 3.3317-4.7460E-4i

2.2500 3.3391-4.7130E-4i

2.2500 3.2148-4.6987E-4i

2.2500 3.1843-4.5897E-4i

2.2500 3.3198-4.3460E-4i

2.2500 3.3426-4.3369E-4i

2.2500 3.3288-4.3141E-4i

2.2500 3.2051-4.3065E-4i

2.2500 3.3343-4.2810E-4i

2.2500 3.2241-4.1822E-4i

2.2500 3.3211-4.1803E-4i

2.2500 3.3233-4.1741E-4i

2.2500 3.3259-4.0855E-4i

2.2500 3.1948-3.9420E-4i

2.2500 3.1843-3.8210E-4i

2.2500 3.3197-3.3134E-4i

2.2500 3.3194-2.7578E-4i

2.2500 3.1882-2.1301E-15i

2.2500 3.1882-1.2255E-14i

2.2500 3.1884+3.9604E-16i

2.2500 3.1884+9.5012E-18i

2.2500 3.2323-2.2040E-16i

2.2500 3.2323+2.0972E-16i

2.2500 3.2338+8.2364E-17i

2.2500 3.2338-1.9012E-16i

2.2500 3.2706+3.9886E-17i

2.2500 3.2706+2.4610E-17i

2.2500 3.2735-1.6123E-16i

2.2500 3.2735-1.1750E-16i

2.2500 3.3030-2.6233E-16i

2.2500 3.3030-7.0712E-17i

2.2500 3.3072-1.9738E-17i

2.2500 3.3072-2.1664E-16i

2.2500 3.3293-1.4399E-16i

2.2500 3.3293-3.0677E-16i

2.2500 3.3348+7.7122E-17i

2.2500 3.3349+1.0991E-17i

2.2500 3.3491-4.6214E-17i

2.2500 3.3491-5.3913E-17i

2.2500 3.3566+1.7731E-16i

2.2500 3.3566+1.3599E-16i

2.2500 3.3619+9.7563E-17i

2.2500 3.3619+9.8402E-17i

2.2500 3.3664-2.6402E-16i

2.2500 3.3724+1.0311E-16i

2.2500 3.3724-1.2642E-16i

2.2500 3.3827+8.5657E-17i

2.2500 3.3827-3.4480E-17i

2.2500 3.3880+7.2165E-17i

2.2500 3.3880+1.8452E-16i

2.2500 3.3895+8.6143E-17i

2.2500 3.3418+0.0019288i

2.2500 3.3445+0.0020178i

2.2500 3.3405+0.0020335i

2.2500 3.3480+0.0020848i

2.2500 3.3709+0.0021006i

2.2500 3.3517+0.0021368i

2.2500 3.3619+0.0021375i

2.2500 3.3553+0.0021799i

2.2500 3.3414+0.0021880i

2.2500 3.3670+0.0022044i

2.2500 3.3646+0.0022283i

2.2500 3.3737+0.0022298i

2.2500 3.3734+0.0022318i

2.2500 3.3444+0.0022332i

2.2500 3.3736+0.0022484i

2.2500 3.3720+0.0022794i

2.2500 3.3478+0.0023041i

2.2500 3.3586+0.0023080i

2.2500 3.3690+0.0023283i

2.2500 3.3586+0.0023483i

2.2500 3.3515+0.0023559i

2.2500 3.3720+0.0023579i

2.2500 3.3730+0.0023855i

2.2500 3.3551+0.0023995i

2.2500 3.3728+0.0024021i

2.2500 3.3689+0.0024231i

2.2500 3.3644+0.0024420i

2.2500 3.3667+0.0024892i

2.2500 3.3615+0.0025137i

2.2500 3.3704+0.0025832i

2.2500 3.2645+0.0026121i

2.2500 3.2896+0.0026731i

2.2500 3.2733+0.0026897i

2.2500 3.1845+0.0027017i

2.2500 3.2969+0.0027281i

2.2500 3.2453+0.0027363i

2.2500 3.3033+0.0027362i

2.2500 3.2351+0.0027373i

2.2500 3.2027+0.0027696i

2.2500 3.2550+0.0027704i

2.2500 3.2136+0.0027777i

2.2500 3.2245+0.0027791i

2.2500 3.2816+0.0028032i

2.2500 3.2550+0.0028368i

2.2500 3.2816+0.0028524i

2.2500 3.2243+0.0028646i

2.2500 3.2451+0.0028703i

2.2500 3.2349+0.0028828i

2.2500 3.2135+0.0029041i

2.2500 3.3087+0.0029116i

2.2500 3.2731+0.0029226i

2.2500 3.1948+0.0029746i

2.2500 3.3136+0.0029818i

2.2500 3.2025+0.0029847i

2.2500 3.3124+0.0029979i

2.2500 3.2642+0.0029989i

2.2500 3.2893+0.0030428i

2.2500 3.2964+0.0030710i

2.2500 3.3122+0.0031768i

2.2500 3.3030+0.0031871i

2.2500 3.3085+0.0031905i

2.3250 3.2508-8.4744E-4i

2.3250 3.2538-8.3956E-4i

2.3250 3.2400-8.3421E-4i

2.3250 3.2460-8.3159E-4i

2.3250 3.2549-8.1861E-4i

2.3250 3.2538-7.6303E-4i

2.3250 3.3338-7.2190E-4i

2.3250 3.2328-7.2114E-4i

2.3250 3.2507-7.0033E-4i

2.3250 3.2068-6.7605E-4i

2.3250 3.3367-6.7042E-4i

2.3250 3.3361-6.6256E-4i

2.3250 3.2161-6.5857E-4i

2.3250 3.2248-6.4765E-4i

2.3250 3.2460-6.4720E-4i

2.3250 3.3300-6.4415E-4i

2.3250 3.3250-6.4290E-4i

2.3250 3.3368-6.2550E-4i

2.3250 3.2248-6.2152E-4i

2.3250 3.3351-6.2156E-4i

2.3250 3.2328-6.1263E-4i

2.3250 3.3276-6.1276E-4i

2.3250 3.3321-6.1293E-4i

2.3250 3.3351-5.9524E-4i

2.3250 3.3190-5.8067E-4i

2.3250 3.3160-5.7875E-4i

2.3250 3.2399-5.7063E-4i

2.3250 3.3366-5.7046E-4i

2.3250 3.3320-5.6806E-4i

2.3250 3.1968-5.6356E-4i

2.3250 3.3221-5.6131E-4i

2.3250 3.3360-5.5641E-4i

2.3250 3.1864-5.5299E-4i

2.3250 3.3131-5.5197E-4i

2.3250 3.2161-5.4975E-4i

2.3250 3.1754-5.4271E-4i

2.3250 3.3107-5.3723E-4i

2.3250 3.3276-5.3149E-4i

2.3250 3.1968-5.3102E-4i

2.3250 3.3220-5.2639E-4i

2.3250 3.3300-5.2160E-4i

2.3250 3.1641-5.1980E-4i

2.3250 3.1863-4.8894E-4i

2.3250 3.3093-4.8263E-4i

2.3250 3.3337-4.7934E-4i

2.3250 3.3190-4.7889E-4i

2.3250 3.3249-4.7489E-4i

2.3250 3.2067-4.7369E-4i

2.3250 3.3107-4.6638E-4i

2.3250 3.3131-4.6401E-4i

2.3250 3.3159-4.5426E-4i

2.3250 3.1754-4.4876E-4i

2.3250 3.1640-4.3560E-4i

2.3250 3.3092-3.6807E-4i

2.3250 3.3088-2.8730E-4i

2.3250 3.1685-6.4672E-14i

2.3250 3.1685-1.2544E-13i

2.3250 3.1687+1.8162E-14i

2.3250 3.1687-3.2070E-14i

2.3250 3.2157+3.9889E-16i

2.3250 3.2157-1.1714E-16i

2.3250 3.2174+3.0460E-17i

2.3250 3.2174+1.8328E-16i

2.3250 3.2568+1.6905E-17i

2.3250 3.2568+1.5678E-17i

2.3250 3.2600+2.9418E-16i

2.3250 3.2600+4.7504E-16i

2.3250 3.2915+3.7289E-16i

2.3250 3.2915+1.8890E-16i

2.3250 3.2961+2.3874E-16i

2.3250 3.2961-1.6071E-16i

2.3250 3.3195-1.5894E-16i

2.3250 3.3195-9.8808E-17i

2.3250 3.3258-2.3773E-16i

2.3250 3.3258-1.4617E-16i

2.3250 3.3407+8.4963E-17i

2.3250 3.3407+6.3024E-17i

2.3250 3.3490+1.6466E-16i

2.3250 3.3491+1.0266E-16i

2.3250 3.3543+1.5130E-16i

2.3250 3.3543+1.8366E-16i

2.3250 3.3591+2.8434E-16i

2.3250 3.3661-3.7678E-16i

2.3250 3.3661-2.9667E-16i

2.3250 3.3771-4.1843E-17i

2.3250 3.3771-1.1591E-18i

2.3250 3.3828+2.8913E-16i

2.3250 3.3828+3.3477E-16i

2.3250 3.3845-4.6813E-17i

2.3250 3.3335+0.0020934i

2.3250 3.3363+0.0021933i

2.3250 3.3320+0.0022073i

2.3250 3.3402+0.0022643i

2.3250 3.3648+0.0022713i

2.3250 3.3551+0.0023148i

2.3250 3.3442+0.0023207i

2.3250 3.3481+0.0023646i

2.3250 3.3331+0.0023778i

2.3250 3.3606+0.0023866i

2.3250 3.3678+0.0024106i

2.3250 3.3675+0.0024116i

2.3250 3.3580+0.0024139i

2.3250 3.3362+0.0024262i

2.3250 3.3676+0.0024313i

2.3250 3.3659+0.0024657i

2.3250 3.3400+0.0025040i

2.3250 3.3516+0.0025041i

2.3250 3.3627+0.0025212i

2.3250 3.3515+0.0025465i

2.3250 3.3659+0.0025515i

2.3250 3.3440+0.0025577i

2.3250 3.3670+0.0025819i

2.3250 3.3669+0.0025999i

2.3250 3.3478+0.0026052i

2.3250 3.3626+0.0026242i

2.3250 3.3578+0.0026462i

2.3250 3.3603+0.0026970i

2.3250 3.3547+0.0027270i

2.3250 3.3642+0.0027981i

2.3250 3.2509+0.0028418i

2.3250 3.2778+0.0028969i

2.3250 3.2604+0.0029211i

2.3250 3.2857+0.0029556i

2.3250 3.2927+0.0029585i

2.3250 3.1649+0.0029819i

2.3250 3.2303+0.0029816i

2.3250 3.2193+0.0029852i

2.3250 3.2407+0.0030162i

2.3250 3.1845+0.0030256i

2.3250 3.2080+0.0030344i

2.3250 3.1962+0.0030353i

2.3250 3.2693+0.0030447i

2.3250 3.2406+0.0030898i

2.3250 3.2692+0.0030947i

2.3250 3.2077+0.0031275i

2.3250 3.2301+0.0031295i

2.3250 3.2191+0.0031460i

2.3250 3.2984+0.0031506i

2.3250 3.1961+0.0031727i

2.3250 3.2601+0.0031781i

2.3250 3.3038+0.0032040i

2.3250 3.3024+0.0032394i

2.3250 3.1761+0.0032442i

2.3250 3.2505+0.0032627i

2.3250 3.1843+0.0032635i

2.3250 3.2775+0.0033011i

2.3250 3.2852+0.0033256i

2.3250 3.3023+0.0034368i

2.3250 3.2922+0.0034517i

2.3250 3.2982+0.0034531i

2.4000 3.2359-9.3461E-4i

2.4000 3.2391-9.2447E-4i

2.4000 3.2243-9.2397E-4i

2.4000 3.2308-9.1844E-4i

2.4000 3.2403-9.0123E-4i

2.4000 3.2391-8.4035E-4i

2.4000 3.2167-7.9988E-4i

2.4000 3.3247-7.9543E-4i

2.4000 3.2358-7.7254E-4i

2.4000 3.1888-7.5422E-4i

2.4000 3.3277-7.3887E-4i

2.4000 3.1988-7.3478E-4i

2.4000 3.3271-7.2970E-4i

2.4000 3.2081-7.2187E-4i

2.4000 3.2307-7.1647E-4i

2.4000 3.3206-7.1028E-4i

2.4000 3.3153-7.0946E-4i

2.4000 3.2081-6.9248E-4i

2.4000 3.3279-6.8858E-4i

2.4000 3.3261-6.8456E-4i

2.4000 3.2166-6.8248E-4i

2.4000 3.3181-6.7558E-4i

2.4000 3.3228-6.7523E-4i

2.4000 3.3260-6.5567E-4i

2.4000 3.3089-6.4137E-4i

2.4000 3.3056-6.3838E-4i

2.4000 3.2242-6.3314E-4i

2.4000 3.1782-6.3222E-4i

2.4000 3.3277-6.2796E-4i

2.4000 3.3228-6.2637E-4i

2.4000 3.1670-6.2118E-4i

2.4000 3.3121-6.2000E-4i

2.4000 3.1988-6.1524E-4i

2.4000 3.3271-6.1225E-4i

2.4000 3.1553-6.1040E-4i

2.4000 3.3025-6.0916E-4i

2.4000 3.1782-5.9652E-4i

2.4000 3.3000-5.9594E-4i

2.4000 3.3181-5.8719E-4i

2.4000 3.1431-5.8432E-4i

2.4000 3.3121-5.8173E-4i

2.4000 3.3206-5.7534E-4i

2.4000 3.1670-5.5131E-4i

2.4000 3.2984-5.3544E-4i

2.4000 3.1888-5.3338E-4i

2.4000 3.3089-5.2957E-4i

2.4000 3.3246-5.2804E-4i

2.4000 3.3152-5.2491E-4i

2.4000 3.3000-5.1826E-4i

2.4000 3.3025-5.1354E-4i

2.4000 3.1552-5.0712E-4i

2.4000 3.3056-5.0305E-4i

2.4000 3.1431-4.9235E-4i

2.4000 3.2984-4.0855E-4i

2.4000 3.2979-2.9634E-4i

2.4000 3.1480+5.3073E-13i

2.4000 3.1480-2.4283E-13i

2.4000 3.1482-3.0430E-13i

2.4000 3.1482+1.2726E-13i

2.4000 3.1986-4.4372E-16i

2.4000 3.1986-2.8407E-16i

2.4000 3.2004-4.7823E-16i

2.4000 3.2004+6.2420E-16i

2.4000 3.2425+3.2360E-16i

2.4000 3.2425+3.2137E-16i

2.4000 3.2460-9.7069E-16i

2.4000 3.2460+2.3800E-16i

2.4000 3.2795+3.5114E-16i

2.4000 3.2795-1.8090E-16i

2.4000 3.2847-2.3732E-17i

2.4000 3.2847+9.8824E-17i

2.4000 3.3095+1.4995E-16i

2.4000 3.3095+2.4497E-17i

2.4000 3.3164+1.1561E-16i

2.4000 3.3164+1.7112E-22i

2.4000 3.3320+1.1143E-16i

2.4000 3.3320+8.3762E-17i

2.4000 3.3413-4.9078E-17i

2.4000 3.3413+1.2268E-17i

2.4000 3.3465-7.9939E-23i

2.4000 3.3465+9.0813E-17i

2.4000 3.3516+2.0671E-16i

2.4000 3.3595+1.8748E-16i

2.4000 3.3595-2.2046E-16i

2.4000 3.3714+8.8372E-17i

2.4000 3.3714+3.6361E-17i

2.4000 3.3775+1.8303E-16i

2.4000 3.3775+5.6515E-17i

2.4000 3.3793+8.4545E-17i

2.4000 3.3249+0.0022652i

2.4000 3.3280+0.0023766i

2.4000 3.3233+0.0023887i

2.4000 3.3586+0.0024481i

2.4000 3.3322+0.0024514i

2.4000 3.3482+0.0024989i

2.4000 3.3364+0.0025126i

2.4000 3.3406+0.0025568i

2.4000 3.3541+0.0025758i

2.4000 3.3245+0.0025761i

2.4000 3.3614+0.0025980i

2.4000 3.3618+0.0025979i

2.4000 3.3512+0.0026066i

2.4000 3.3616+0.0026210i

2.4000 3.3279+0.0026277i

2.4000 3.3598+0.0026589i

2.4000 3.3445+0.0027081i

2.4000 3.3319+0.0027127i

2.4000 3.3563+0.0027215i

2.4000 3.3444+0.0027525i

2.4000 3.3598+0.0027524i

2.4000 3.3363+0.0027678i

2.4000 3.3609+0.0027856i

2.4000 3.3608+0.0028051i

2.4000 3.3404+0.0028194i

2.4000 3.3562+0.0028329i

2.4000 3.3510+0.0028583i

2.4000 3.3537+0.0029129i

2.4000 3.3477+0.0029490i

2.4000 3.3579+0.0030211i

2.4000 3.2369+0.0030824i

2.4000 3.2657+0.0031298i

2.4000 3.2470+0.0031628i

2.4000 3.2817+0.0031887i

2.4000 3.2742+0.0031924i

2.4000 3.2148+0.0032391i

2.4000 3.2030+0.0032458i

2.4000 3.2260+0.0032740i

2.4000 3.1446+0.0032802i

2.4000 3.1657+0.0032953i

2.4000 3.2566+0.0032970i

2.4000 3.1909+0.0033033i

2.4000 3.1782+0.0033068i

2.4000 3.2565+0.0033474i

2.4000 3.2259+0.0033552i

2.4000 3.2879+0.0033988i

2.4000 3.2146+0.0034017i

2.4000 3.1906+0.0034042i

2.4000 3.2028+0.0034229i

2.4000 3.2939+0.0034303i

2.4000 3.2467+0.0034453i

2.4000 3.1781+0.0034556i

2.4000 3.2923+0.0034906i

2.4000 3.1567+0.0035279i

2.4000 3.2365+0.0035387i

2.4000 3.1655+0.0035575i

2.4000 3.2654+0.0035703i

2.4000 3.2737+0.0035898i

2.4000 3.2921+0.0037078i

2.4000 3.2877+0.0037256i

2.4000 3.2812+0.0037263i
