# Supplementary material for: Theoretical proposal of a low-loss wide-bandwidth silicon photonic crystal fiber for supporting 30 orbital angular momentum modes
Source: PLoS One. 2017 Dec 13;12(12):e0189660. doi: 10.1371/journal.pone.0189660 (PMC5728573; doi:10.1371/journal.pone.0189660)
Supplement: S1 Table — (PDF) [file pone.0189660.s002.PDF]

	EH71	HE91	EH61	HE81	EH51	HE71	EH41	HE61
1.2	3.3942	3.3943	3.4063	3.4065	3.4169	3.4172	3.426	3.4263
1.275	3.3837	3.3838	3.3974	3.3976	3.4094	3.4097	3.4196	3.4201
1.35	3.3725	3.3726	3.3879	3.3882	3.4014	3.4018	3.4129	3.4135
1.425	3.3608	3.3609	3.3779	3.3782	3.393	3.3935	3.4057	3.4065
1.5	3.34834	3.34846	3.3674	3.3678	3.3841	3.3847	3.3983	3.3992
1.575	3.33528	3.33542	3.3563	3.3568	3.3748	3.3755	3.3904	3.3915
1.65	3.32157	3.32173	3.3447	3.3453	3.365	3.3659	3.3822	3.3835
1.725	3.30722	3.3074	3.3326	3.3332	3.3548	3.3558	3.3736	3.3751
1.8	3.29221	3.29241	3.3199	3.3206	3.3441	3.3454	3.3646	3.3664
1.875	3.27655	3.27676	3.3067	3.3075	3.333	3.3345	3.3553	3.3574
1.95	3.26022	3.26045	3.2929	3.2938	3.3214	3.3231	3.3456	3.348
2.025	3.24323	3.24347	3.2786	3.2797	3.3094	3.3114	3.3355	3.3383
2.1	3.22556	3.22581	3.2637	3.2649	3.2969	3.2992	3.3251	3.3283
2.175	3.20722	3.20746	3.2483	3.2496	3.284	3.2865	3.3142	3.3179
2.25	3.18818	3.18842	3.2323	3.2338	3.2706	3.2735	3.303	3.3072
2.325	3.16846	3.16867	3.2157	3.2174	3.2568	3.26	3.2915	3.2961
2.4	3.14803	3.14821	3.1986	3.2004	3.2425	3.246	3.2795	3.2847

EH31	HE51	EH21	HE41	EH11	TM01	HE31	HE21	HE11
3.4334	3.4339	3.4391	3.4398	3.4429	3.4444	3.4441	3.4468	3.448
3.428	3.4286	3.4344	3.4353	3.4387	3.4404	3.4402	3.4432	3.4446
3.4222	3.4231	3.4294	3.4306	3.4343	3.4363	3.4361	3.4395	3.4411
3.4162	3.4172	3.4242	3.4256	3.4295	3.4315	3.4317	3.4356	3.4374
3.4098	3.4111	3.4187	3.4204	3.4246	3.4268	3.4272	3.4315	3.4335
3.4032	3.4047	3.4129	3.415	3.4194	3.4218	3.4225	3.4273	3.4296
3.3962	3.398	3.4069	3.4094	3.414	3.4166	3.4176	3.4229	3.4255
3.3889	3.391	3.4006	3.4035	3.4083	3.4111	3.4126	3.4184	3.4212
3.3813	3.3838	3.3941	3.3974	3.4024	3.4054	3.4074	3.4137	3.4168
3.3734	3.3763	3.3872	3.3911	3.3962	3.3995	3.4019	3.4089	3.4123
3.3652	3.3685	3.3801	3.3846	3.3898	3.3933	3.3964	3.4039	3.4077
3.3567	3.3605	3.3728	3.3779	3.3832	3.3869	3.3906	3.3988	3.403
3.3479	3.3522	3.3652	3.371	3.3763	3.3803	3.3847	3.3936	3.3981
3.3387	3.3437	3.3573	3.3639	3.3692	3.3735	3.3787	3.3882	3.3931
3.3293	3.3348	3.3491	3.3566	3.3619	3.3664	3.3724	3.3827	3.388
3.3195	3.3258	3.3407	3.349	3.3543	3.3591	3.3661	3.3771	3.3828
3.3095	3.3164	3.332	3.3413	3.3465	3.3516	3.3595	3.3714	3.3775

TE01
3.4482
3.4448
3.4414
3.4378
3.434
3.4301
3.4261
3.4219
3.4176
3.4132
3.4087
3.4041
3.3993
3.3945
3.3895
3.3845
3.3793
