# Supplementary material for: Theoretical proposal of a low-loss wide-bandwidth silicon photonic crystal fiber for supporting 30 orbital angular momentum modes
Source: PLoS One. 2017 Dec 13;12(12):e0189660. doi: 10.1371/journal.pone.0189660 (PMC5728573; doi:10.1371/journal.pone.0189660)
Supplement: S2 Table — (PDF) [file pone.0189660.s003.pdf]

	HE91-EH71	HE81-EH61	HE71-EH51	HE61-EH41	HE51-EH31	HE41-EH21	HE31-EH11
1.2	1E-04	0.0002	0.0003	0.0003	0.0005	0.0007	0.0012
1.275	1E-04	0.0002	0.0003	0.0005	0.0006	0.0009	0.0015
1.35	1E-04	0.0003	0.0004	0.0006	0.0009	0.0012	0.0018
1.425	0.0001	0.0003	0.0005	0.0008	0.001	0.0014	0.0022
1.5	0.00012	0.0004	0.0006	0.0009	0.0013	0.0017	0.0026
1.575	0.00014	0.0005	0.0007	0.0011	0.0015	0.0021	0.0031
1.65	0.00016	0.0006	0.0009	0.0013	0.0018	0.0025	0.0036
1.725	0.00018	0.0006	0.001	0.0015	0.0021	0.0029	0.0043
1.8	0.0002	0.0007	0.0013	0.0018	0.0025	0.0033	0.005
1.875	0.00021	0.0008	0.0015	0.0021	0.0029	0.0039	0.0057
1.95	0.00023	0.0009	0.0017	0.0024	0.0033	0.0045	0.0066
2.025	0.00024	0.0011	0.002	0.0028	0.0038	0.0051	0.0074
2.1	0.00025	0.0012	0.0023	0.0032	0.0043	0.0058	0.0084
2.175	0.00024	0.0013	0.0025	0.0037	0.005	0.0066	0.0095
2.25	0.00024	0.0015	0.0029	0.0042	0.0055	0.0075	0.0105
2.325	0.00021	0.0017	0.0032	0.0046	0.0063	0.0083	0.0118
2.4	0.00018	0.0018	0.0035	0.0052	0.0069	0.0093	0.013

HE21-TM01	TE01-HE21
0.0024	0.0014
0.0028	0.0016
0.0032	0.0019
0.0041	0.0022
0.0047	0.0025
0.0055	0.0028
0.0063	0.0032
0.0073	0.0035
0.0083	0.0039
0.0094	0.0043
0.0106	0.0048
0.0119	0.0053
0.0133	0.0057
0.0147	0.0063
0.0163	0.0068
0.018	0.0074
0.0198	0.0079
