# Supplementary material for: Theoretical proposal of a low-loss wide-bandwidth silicon photonic crystal fiber for supporting 30 orbital angular momentum modes
Source: PLoS One. 2017 Dec 13;12(12):e0189660. doi: 10.1371/journal.pone.0189660 (PMC5728573; doi:10.1371/journal.pone.0189660)
Supplement: S3 Table — (PDF) [file pone.0189660.s004.pdf]

	EH71	HE91	EH61	HE81	EH51	HE71	EH41	HE61
1.2	9.18E-17	7.34E-17	2.34E-17	8.39E-18	6.95E-17	2.19E-18	5.46E-17	8.69E-17
1.275	6.03E-17	3.29E-17	8.49E-17	9.88E-18	2E-17	1.08E-17	7.52E-17	7.17E-17
1.35	1.81E-16	1.64E-17	6.25E-17	2.24E-17	1.49E-17	6.49E-17	4.89E-17	2.45E-16
1.425	1.48E-16	1.56E-16	7.85E-17	7.04E-17	4.47E-17	5.53E-19	3.85E-17	1.71E-16
1.5	1.09E-16	2.28E-17	1.2E-16	4.99E-17	5.18E-17	2.04E-17	6.43E-18	3.32E-17
1.575	9.23E-17	1.06E-16	4.43E-17	2.44E-16	1.13E-17	6.41E-17	8.76E-17	1.91E-17
1.65	1.52E-16	1.83E-16	1.21E-16	1.17E-17	1.8E-17	1.63E-16	1.74E-16	6.2E-17
1.725	1.24E-16	5.42E-16	5.16E-16	3.14E-16	1.26E-16	1.59E-16	6.66E-17	6.08E-17
1.8	1.99E-16	1.19E-16	1.37E-16	4.55E-16	1.41E-16	3.06E-16	1.32E-18	7.02E-17
1.875	1.08E-16	8.97E-17	3.12E-16	3.06E-16	2.34E-17	5.66E-18	2.63E-17	1.3E-16
1.95	8.52E-17	6.31E-17	2.77E-16	1.68E-16	1.36E-16	1.02E-16	2.02E-16	1.27E-16
2.025	8.13E-17	1.19E-16	2.05E-16	2.49E-16	3.07E-17	1.43E-17	3.87E-17	1.17E-16
2.1	8.69E-17	3.24E-16	1.93E-16	1.73E-16	1.71E-16	1.51E-16	5.77E-17	6.03E-17
2.175	8.38E-16	2.61E-16	7.38E-18	2.19E-16	1.43E-16	3.07E-16	5.11E-17	2.7E-17
2.25	2.13E-15	3.96E-16	2.2E-16	8.24E-17	2.46E-17	1.61E-16	2.62E-16	2.17E-16
2.325	6.47E-14	1.82E-14	3.99E-16	3.05E-17	1.57E-17	2.94E-16	3.73E-16	1.61E-16
2.4	5.31E-13	3.04E-13	4.44E-16	4.78E-16	3.21E-16	9.71E-16	3.51E-16	9.88E-17

EH31	HE51	EH21	HE41	EH11	HE31	HE21	HE11
3E-17	2.82E-17	1.36E-17	6.93E-17	1.93E-17	4.11E-18	3.04E-18	7.79E-17
2.38E-18	3.78E-17	4.02E-17	3.83E-17	2.37E-17	8.38E-18	2.48E-17	4.65E-17
8.51E-17	7.16E-17	2.5E-17	3.71E-17	2.69E-17	4.05E-18	2.3E-17	7.82E-18
5.45E-18	1.08E-16	1.58E-17	5.15E-17	1.05E-16	2.29E-17	2.54E-17	7.11E-17
3.89E-17	2.14E-17	7.63E-17	8.04E-19	1.37E-16	4.83E-17	1.87E-17	8.31E-18
5.72E-17	2.59E-17	5.69E-18	3.04E-17	8.33E-17	1.76E-16	4.5E-17	1.02E-17
6.33E-17	7.31E-17	4.26E-17	2.89E-17	3.9E-17	5.26E-17	2.05E-17	4.3E-17
6.66E-17	5.06E-17	7.05E-17	4.56E-17	9.26E-17	1.19E-16	2.1E-17	7.1E-17
8.12E-17	1.03E-16	2.19E-17	1.08E-16	1.01E-16	1.45E-16	5.03E-18	1.03E-16
1.09E-16	1.47E-16	5.57E-17	5.46E-17	1.35E-17	2.46E-17	3.96E-17	6.13E-17
1.08E-16	9.82E-17	9.4E-18	6.12E-17	1.23E-16	1.38E-16	1.09E-16	1.09E-17
2.97E-17	6.31E-17	2.26E-16	8.55E-17	6.6E-17	1.7E-16	2.85E-17	6.1E-17
1E-16	1.04E-16	8E-17	1.84E-16	4.43E-17	1.02E-16	1.38E-16	2.06E-16
5.59E-17	7.91E-17	3.48E-18	4.12E-16	1.68E-17	3.75E-17	6.83E-17	1.03E-16
1.44E-16	7.71E-17	5.39E-17	1.36E-16	9.84E-17	1.26E-16	8.57E-17	7.22E-17
1.59E-16	2.38E-16	6.3E-17	1.03E-16	1.84E-16	2.97E-16	4.18E-17	2.89E-16
1.5E-16	1.16E-16	8.38E-17	1.23E-17	9.08E-17	2.2E-16	8.84E-17	1.83E-16
