# Supplementary material for: Theoretical proposal of a low-loss wide-bandwidth silicon photonic crystal fiber for supporting 30 orbital angular momentum modes
Source: PLoS One. 2017 Dec 13;12(12):e0189660. doi: 10.1371/journal.pone.0189660 (PMC5728573; doi:10.1371/journal.pone.0189660)
Supplement: S4 Table — (PDF) [file pone.0189660.s005.pdf]

	EH71	HE91	EH61	HE81	EH51	HE71	EH41	HE61
1.2	4.17E-09	3.34E-09	1.06E-09	3.82E-10	3.16E-09	9.95E-11	2.48E-09	3.95E-09
1.275	2.58E-09	1.41E-09	3.63E-09	4.23E-10	8.57E-10	4.63E-10	3.22E-09	3.07E-09
1.35	7.32E-09	6.64E-10	2.52E-09	9.06E-10	6E-10	2.62E-09	1.98E-09	9.92E-09
1.425	5.66E-09	5.97E-09	3.01E-09	2.7E-09	1.71E-09	2.12E-11	1.47E-09	6.56E-09
1.5	3.95E-09	8.29E-10	4.35E-09	1.81E-09	1.88E-09	7.44E-10	2.34E-10	1.21E-09
1.575	3.2E-09	3.66E-09	1.54E-09	8.45E-09	3.93E-10	2.22E-09	3.03E-09	6.63E-10
1.65	5.04E-09	6.06E-09	4E-09	3.88E-10	5.96E-10	5.38E-09	5.75E-09	2.05E-09
1.725	3.92E-09	1.71E-08	1.63E-08	9.93E-09	3.99E-09	5.04E-09	2.11E-09	1.92E-09
1.8	6.03E-09	3.6E-09	4.15E-09	1.38E-08	4.26E-09	9.28E-09	4.01E-11	2.13E-09
1.875	3.15E-09	2.61E-09	9.07E-09	8.91E-09	6.81E-10	1.65E-10	7.67E-10	3.77E-09
1.95	2.38E-09	1.76E-09	7.76E-09	4.7E-09	3.79E-09	2.85E-09	5.65E-09	3.56E-09
2.025	2.19E-09	3.2E-09	5.53E-09	6.71E-09	8.27E-10	3.86E-10	1.04E-09	3.15E-09
2.1	2.26E-09	8.43E-09	5.02E-09	4.49E-09	4.46E-09	3.93E-09	1.5E-09	1.57E-09
2.175	2.1E-08	6.54E-09	1.85E-10	5.49E-09	3.58E-09	7.7E-09	1.28E-09	6.78E-10
2.25	5.17E-08	9.61E-09	5.35E-09	2E-09	5.97E-10	3.91E-09	6.36E-09	5.25E-09
2.325	1.52E-06	4.26E-07	9.36E-09	7.15E-10	3.68E-10	6.91E-09	8.75E-09	3.77E-09
2.4	1.21E-05	6.92E-06	1.01E-08	1.09E-08	7.31E-09	2.21E-08	7.98E-09	2.25E-09

EH31	HE51	EH21	HE41	EH11	HE31	HE21	HE11
1.36E-09	1.28E-09	6.19E-10	3.15E-09	8.77E-10	1.87E-10	1.38E-10	3.54E-09
1.02E-10	1.62E-09	1.72E-09	1.64E-09	1.01E-09	3.59E-10	1.06E-09	1.99E-09
3.44E-09	2.89E-09	1.01E-09	1.5E-09	1.09E-09	1.64E-10	9.28E-10	3.16E-10
2.09E-10	4.14E-09	6.04E-10	1.97E-09	4.01E-09	8.79E-10	9.74E-10	2.72E-09
1.42E-09	7.79E-10	2.78E-09	2.92E-11	4.99E-09	1.76E-09	6.82E-10	3.02E-10
1.98E-09	8.98E-10	1.97E-10	1.05E-09	2.89E-09	6.11E-09	1.56E-09	3.52E-10
2.09E-09	2.42E-09	1.41E-09	9.56E-10	1.29E-09	1.74E-09	6.79E-10	1.42E-09
2.11E-09	1.6E-09	2.23E-09	1.44E-09	2.93E-09	3.76E-09	6.64E-10	2.25E-09
2.46E-09	3.12E-09	6.64E-10	3.28E-09	3.06E-09	4.39E-09	1.53E-10	3.12E-09
3.17E-09	4.27E-09	1.62E-09	1.59E-09	3.93E-10	7.15E-10	1.15E-09	1.79E-09
3.02E-09	2.75E-09	2.63E-10	1.71E-09	3.43E-09	3.87E-09	3.04E-09	3.05E-10
8E-10	1.7E-09	6.09E-09	2.31E-09	1.78E-09	4.57E-09	7.67E-10	1.64E-09
2.6E-09	2.71E-09	2.08E-09	4.78E-09	1.15E-09	2.66E-09	3.57E-09	5.34E-09
1.4E-09	1.99E-09	8.73E-11	1.03E-08	4.22E-10	9.41E-10	1.71E-09	2.57E-09
3.49E-09	1.87E-09	1.31E-09	3.3E-09	2.39E-09	3.07E-09	2.08E-09	1.75E-09
3.73E-09	5.58E-09	1.48E-09	2.41E-09	4.31E-09	6.96E-09	9.82E-10	6.79E-09
3.41E-09	2.63E-09	1.9E-09	2.79E-10	2.07E-09	5.01E-09	2.01E-09	4.16E-09
