# Supplementary material for: Theoretical proposal of a low-loss wide-bandwidth silicon photonic crystal fiber for supporting 30 orbital angular momentum modes
Source: PLoS One. 2017 Dec 13;12(12):e0189660. doi: 10.1371/journal.pone.0189660 (PMC5728573; doi:10.1371/journal.pone.0189660)
Supplement: S5 Table — (PDF) [file pone.0189660.s006.pdf]

	EH61	HE81	EH51	HE71	EH41	HE61	EH31	HE51
1.425	-660.41	-658.44	-727.59	-749.1	-796.82	-803.16	-732.9	-763.24
1.5	-422.19	-422.61	-495.69	-517.37	-567.94	-583.11	-500.63	-540.95
1.575	-250.05	-252.57	-330.13	-351.88	-405.37	-428.69	-334.89	-384.4
1.65	-113.83	-118.11	-200.78	-222.51	-278.98	-309.55	-205.59	-263.24
1.725	-0.43749	-6.0479	-94.534	-116.14	-175.68	-212.38	-99.65	-164.19
1.8	97.024	90.579	-4.494	-25.882	-88.574	-130.05	-10.217	-80.146
1.875	182.71	176	73.495	52.42	-13.506	-58.207	66.832	-6.7513
1.95	259.37	253.05	142.18	121.51	52.261	6.1159	134.2	58.934
2.025	328.93	323.72	203.49	183.33	110.66	65.064	193.8	119.04
2.1	392.82	389.51	258.85	239.29	163.09	120.27	247	175.19
2.175	452.12	451.58	309.33	290.47	210.65	173.04	294.84	228.65
2.25	507.65	510.84	355.78	337.72	254.16	224.41	338.14	280.46
2.325	560.09	568.02	398.86	381.68	294.28	275.26	377.52	331.49
2.4	609.97	623.73	439.1	422.91	331.53	326.35	413.46	382.45

EH21	HE41	EH11	HE31	HE21	HE11
-786.85	-829.1	-808.63	-880.55	-913.62	-932.9
-558.17	-610.39	-583.27	-663.09	-695.4	-718.99
-395.52	-457.58	-424.44	-511.95	-544.58	-571.43
-268.71	-340.37	-301.99	-396.93	-431.12	-459.98
-164.61	-245.47	-202.81	-304.82	-342.01	-371.43
-76.263	-165.82	-120	-228.64	-270.45	-298.79
0.52992	-97.094	-49.4	-164.13	-212.38	-237.79
68.56	-36.36	11.748	-108.45	-165.15	-185.6
129.81	18.485	65.379	-59.595	-126.93	-140.17
185.73	69.036	112.92	-16.036	-96.38	-99.977
237.47	116.55	155.47	23.395	-72.527	-63.849
285.9	162.03	193.85	59.627	-54.625	-30.842
331.73	206.32	228.75	93.415	-42.107	-0.19087
375.54	250.13	260.69	125.38	-34.538	28.738
