# Supplementary material for: Theoretical proposal of a low-loss wide-bandwidth silicon photonic crystal fiber for supporting 30 orbital angular momentum modes
Source: PLoS One. 2017 Dec 13;12(12):e0189660. doi: 10.1371/journal.pone.0189660 (PMC5728573; doi:10.1371/journal.pone.0189660)
Supplement: S6 Table — (PDF) [file pone.0189660.s007.pdf]

| | HE81-EH61 | HE71-EH51 | HE61-EH41 | HE51-EH31 | HE41-EH21 | HE31-EH11 | HE21-TM01 |
|---|---|---|---|---|---|---|---|
| 1.2 | 0.0002 | 0.0002 | 0.0003 | 0.0005 | 0.0006 | 0.001 | 0.0022 |
| 1.275 | 1E-04 | 0.0002 | 0.0004 | 0.0006 | 0.0008 | 0.0013 | 0.0026 |
| 1.35 | 0.0002 | 0.0004 | 0.0005 | 0.0007 | 0.00105 | 0.00155 | 0.0031 |
| 1.425 | 0.0003 | 0.0005 | 0.0007 | 0.0009 | 0.0013 | 0.0019 | 0.0037 |
| 1.5 | 0.0003 | 0.0005 | 0.0008 | 0.001 | 0.0015 | 0.0023 | 0.0043 |
| 1.575 | 0.0004 | 0.0006 | 0.0009 | 0.0012 | 0.0018 | 0.0028 | 0.0051 |
| 1.65 | 0.0004 | 0.0008 | 0.0011 | 0.0016 | 0.0021 | 0.0033 | 0.0059 |
| 1.725 | 0.0005 | 0.0009 | 0.0013 | 0.0018 | 0.0026 | 0.0038 | 0.0067 |
| 1.8 | 0.0006 | 0.001 | 0.0015 | 0.0021 | 0.0029 | 0.0044 | 0.0076 |
| 1.875 | 0.0006 | 0.0012 | 0.0017 | 0.0025 | 0.0034 | 0.0051 | 0.0086 |
| 1.95 | 0.0008 | 0.0013 | 0.002 | 0.0028 | 0.0039 | 0.0058 | 0.0097 |
| 2.025 | 0.0009 | 0.0015 | 0.0023 | 0.0033 | 0.0044 | 0.0066 | 0.0108 |
| 2.1 | 0.001 | 0.0017 | 0.0026 | 0.0037 | 0.005 | 0.0074 | 0.0121 |
| 2.175 | 0.0011 | 0.0019 | 0.003 | 0.0042 | 0.0058 | 0.0083 | 0.0134 |
| 2.25 | 0.0012 | 0.0022 | 0.0034 | 0.0046 | 0.0065 | 0.0093 | 0.0148 |
| 2.325 | 0.0013 | 0.0025 | 0.0038 | 0.0052 | 0.0072 | 0.0103 | 0.0163 |
| 2.4 | 0.0015 | 0.0027 | 0.0042 | 0.0059 | 0.008 | 0.0114 | 0.0179 |

| TE01-HE21 |
| ---: |
| 0.0013 |
| 0.0015 |
| 0.0018 |
| 0.0021 |
| 0.0024 |
| 0.0026 |
| 0.003 |
| 0.0034 |
| 0.0038 |
| 0.0041 |
| 0.0046 |
| 0.005 |
| 0.0055 |
| 0.006 |
| 0.0065 |
| 0.007 |
| 0.0076 |
