# Supplementary material for: Theoretical proposal of a low-loss wide-bandwidth silicon photonic crystal fiber for supporting 30 orbital angular momentum modes
Source: PLoS One. 2017 Dec 13;12(12):e0189660. doi: 10.1371/journal.pone.0189660 (PMC5728573; doi:10.1371/journal.pone.0189660)
Supplement: S8 Table — (PDF) [file pone.0189660.s009.pdf]

	EH61	HE81	EH51	HE71	EH41	HE61	EH31	HE51
1.425	-473.77	-485.41	-590.33	-596.45	-677.87	-691.13	-735.79	-779.35
1.5	-227.09	-237.48	-347.11	-357.44	-438.73	-461.56	-510.89	-551.06
1.575	-46.239	-55.644	-170.35	-184.91	-266.71	-298.05	-351.47	-389.8
1.65	98.972	90.205	-29.996	-48.747	-131.86	-170.35	-227.17	-265.53
1.725	221.69	213.16	86.982	64.121	-21.249	-65.27	-124.68	-165.21
1.8	328.88	320.1	187.41	160.57	71.839	24.203	-36.886	-82.048
1.875	424.73	415.18	275.35	244.74	151.38	102.32	40.594	-11.967
1.95	512.06	501.12	353.5	319.35	219.95	171.93	110.72	47.693
2.025	592.83	579.85	423.69	386.3	279.29	235.07	175.64	98.778
2.1	668.54	652.77	487.29	447	330.66	293.27	236.99	142.62
2.175	740.3	720.95	545.28	502.51	374.95	347.69	296.07	180.22
2.25	809.02	785.21	598.44	553.63	412.82	399.29	353.94	212.33
2.325	875.4	846.21	647.34	601.01	444.77	448.82	411.47	239.52
2.4	940.03	904.45	692.46	645.15	471.13	496.93	469.42	262.24

EH21	HE41	EH11	HE31	HE21	HE11
-797.69	-829.84	-819.28	-879.36	-915.24	-930.36
-567.18	-612.24	-598.24	-663.11	-700.31	-717.84
-404.01	-460.71	-442.98	-513.04	-551.66	-571.71
-278.21	-344.95	-323.18	-398.88	-439.07	-461.73
-176.85	-251.69	-225.58	-307.39	-349.3	-374.67
-93.217	-173.87	-143.12	-231.54	-275.37	-303.52
-23.324	-107.16	-71.461	-167.04	-213	-244
35.399	-48.652	-7.6987	-111.01	-159.34	-193.26
84.71	3.7502	50.269	-61.392	-112.36	-149.25
125.86	51.637	104.03	-16.624	-70.508	-110.42
159.74	96.252	154.84	24.505	-32.612	-75.581
187.03	138.6	203.69	62.964	2.2779	-43.779
208.21	179.51	251.42	99.552	34.934	-14.233
223.64	219.68	298.72	134.93	65.995	13.705
