# Supplementary material for: Theoretical proposal of a low-loss wide-bandwidth silicon photonic crystal fiber for supporting 30 orbital angular momentum modes
Source: PLoS One. 2017 Dec 13;12(12):e0189660. doi: 10.1371/journal.pone.0189660 (PMC5728573; doi:10.1371/journal.pone.0189660)
Supplement: S9 Table — (PDF) [file pone.0189660.s010.pdf]

	EH51	HE71	EH41	HE61	EH31	HE51	EH21	HE41
1.425	-590.33	-596.45	-677.87	-687.96	-735.79	-779.35	-797.69	-831.43
1.5	-347.11	-357.44	-438.73	-453.92	-510.89	-551.06	-567.18	-612.42
1.575	-170.35	-184.91	-266.71	-290.66	-351.47	-389.8	-404.01	-459.78
1.65	-29.996	-48.747	-131.86	-166.66	-227.17	-265.53	-278.21	-343.27
1.725	86.982	64.121	-21.249	-67.054	-124.68	-165.21	-176.85	-249.69
1.8	187.41	160.57	71.839	17.188	-36.886	-82.048	-93.217	-172.04
1.875	275.35	244.74	151.38	92.719	40.594	-11.967	-23.324	-106.06
1.95	353.5	319.35	219.95	165.15	110.72	47.693	35.399	-48.906
2.025	423.69	386.3	279.29	239.66	175.64	98.778	84.71	1.4608
2.1	487.29	447	330.66	321.26	236.99	142.62	125.86	46.562
2.175	545.28	502.51	374.95	415.01	296.07	180.22	159.74	87.579
2.25	598.44	553.63	412.82	526.09	353.94	212.33	187.03	125.45
2.325	647.34	601.01	444.77	659.88	411.47	239.52	208.21	160.94
2.4	692.46	645.15	471.13	821.98	469.42	262.24	223.64	194.69

EH11	HE31	HE21	HE11
-819.28	-879.36	-915.24	-928.79
-598.24	-663.11	-700.31	-717.68
-442.98	-513.04	-551.66	-572.67
-323.18	-398.88	-439.07	-463.44
-225.58	-307.39	-349.3	-376.7
-143.12	-231.54	-275.37	-305.37
-71.461	-167.04	-213	-245.13
-7.6987	-111.01	-159.34	-193.04
50.269	-61.392	-112.36	-146.99
104.03	-16.624	-70.508	-105.38
154.84	24.505	-32.612	-66.942
203.69	62.964	2.2779	-30.665
251.42	99.552	34.934	4.2986
298.72	134.93	65.995	38.66
